# Activated carbon for aerobic oxidation: Benign approach toward 2-benzoylbenzimidazoles and 2-benzoylbenzoxazoles synthesis

**DOI:** 10.1038/srep10360

**Published:** 2015-06-04

**Authors:** Kai Bao, Fuqing Li, Hanjing Liu, Zhiwei Wang, Qirong Shen, Jian Wang, Weige Zhang

**Affiliations:** 1 Key Laboratory of Structure-Based Drug Design & Discovery, Ministry of Education Shenyang Pharmaceutical University, Shenyang 110016 (China); 2 Division of Hematology/Oncology, Department of Medicine, Beth Israel Deaconess Medical Center, Harvard Medical School, Boston, MA 02215 (USA)

## Abstract

A general strategy involving a novel and highly efficient aerobic benzylic oxidation promoted by cheap, reusable activated carbon in water is developed. Application of this method has been demonstrated in the benign synthesis of bioactive 2-benzoylbenzimidazoles and 2-benzoylbenzoxazoles derivatives. Furthermore, the activated carbon catalyst could be recovered and reused at least three times without significantly losing its activity. Preliminary research suggests that the oxidation mechanism may involve intermediate hydroperoxidation and that a portion of the final carbonyl product is obtained through a secondary benzylic alcohol intermediate. Finally, theoretical calculations reveal that the oxidation yield is closely associated with the electric density at the benzylic position of the substrate.

Benzylic oxidation is of considerable importance in both laboratory and industrial syntheses. Large quantities of oxidants including Mn^VII^, Cr^VI^ and I^V^ have traditionally been used for benzylic oxidation, but each results in large amounts of unwanted waste^1^,^2^,^3^. Molecular oxygen is a safe, inexpensive, and environmentally friendly oxidant. Typically, benzylic oxidation with O_2_ is carried out under extreme conditions, such as high temperature or irradiation, thus forming a free radical or singlet oxygen^4^,^5^. However, most of aerobic benzylic oxidations that involve milder conditions may use expensive and/or toxic transition metals, such as Pd, Ru and Rh^6^,^7^,^8^,^9^.

Carbon chemistry is one of the most active and promising research fields in organic synthesis and catalyst research. Recent reports have shown an outstanding performance of nanoscaled carbon catalysts in several important reactions and related chemical industrial processes^10^,^11^,^12^,^13^,^14^. On the other hand, activated carbon (AC) is widely used as a low-cost adsorbent in the chemical industry. A new promising method has been reported using AC/O_2_ system for oxidation of benzylic alcohols or alkylarenes (fluorenes, xanthenes and anthrones) to the corresponding carbonyl compounds^15^,^16^. It is worth noting that using nonpolar *m*-xylene at relative high temperature is recommended in these methods.

The use of water as the reaction solvent becomes an attractive research area in green chemistry^17^,^18^,^19^,^20^. Although much progress has been made in the field of aqueous organic oxidations, only a few examples of benzylic oxidation performed in water by stoichiometric hypervalent iodine or photocatalysis have been described so far^21^,^22^,^23^. Herein, we wish to report an activated carbon-promoted aerobic benzylic oxidation in water as a green synthetic approach to 2-benzoylbenzimidazoles and 2-benzoylbenzoxazoles.

## Results and Discussion

**Figure 1:**
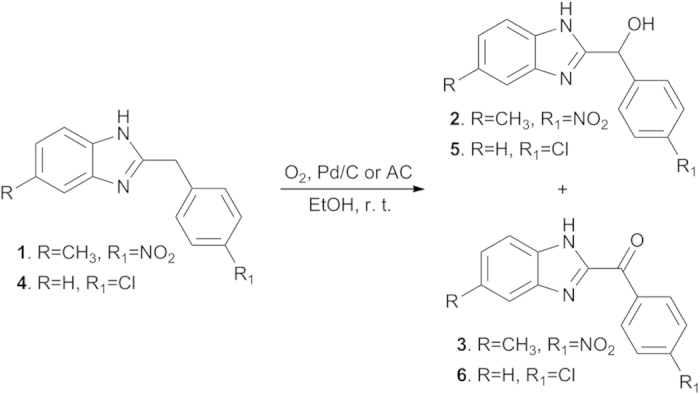
The aerobic benzylic oxidation of 2-benzylbenzo[d]imidazoles.

During the intended preparation of 5-methyl-2-(4-azylbenzyl)benzo[d]imidazole *via* catalytic hydrogenation for the evaluation of its bioactivity, 5-methyl-2-(4-nitrobenzyl)benzo[d]imidazole (**1**) was inadvertently placed in a mixture of 10% Pd/C in ethanol under air at room temperature for approximately 12 h. The majority of the starting material remained unreacted, however, two unexpected products, 5-methyl-2-(4-nitrohydroxybenzyl)benzo[d]imidazole (**2**) and 5-methyl-2-(4-nitrobenzoyl)benzo[d]imidazole (**3**), were detected. This reaction was checked using 2-(4-chlorobenzyl)benzo[d]imidazole (**4**) as the substrate, and two oxidation products, **5** and **6**, were obtained (Fig. 1). These results showed that the benzylic methylenes of 2-benzoylbenzo[d]imidazoles could be oxidized in air in the presence of Pd/C.

To explore the role of Pd/C in this reaction, compound **1** and **4** were treated in ethanol in the absence of Pd/C under air at room temperature for 24 h respectively, and no oxidation products were observed. The reactions were then carried out utilizing various Pd^0^ or Pd^II^ species, including Pd black, Pd[PPh_3_]_4_, Pd(OAc)_2_, PdCl_2_ and [Ph_3_P]_2_PdCl_2_, as the catalysts. However, no desired product could be detected in the reaction mixture after 24 h. On the other hand, the Pd leaching from 10% Pd/C during the oxidation of **4** was measured and no leached palladium species was detected after the phase separation of the reaction mixture (Shimadzu, Kyoto, Japan, AA-7000, <1 ppm detection limit)^24^. These unexpected results prompted us to examine the effectiveness of activated carbon in the oxidation.

Using commercially available AC to replace Pd/C, the reaction was repeated and the benzylic oxidation products were clearly achieved after 6 h. Three types of AC were tested and the desired products were obtained respectively, supporting the effectivity of AC in these reactions. The results indicated that activated carbon, instead of palladium, was crucial for the aerobic benzylic oxidation of 2-benzylbenzo[d]imidazoles.

2-(4-Chlorobenzoyl)benzo[d]imidazole (**4**) was then chosen as the model substrate to examine the influence of atmosphere, temperature and solvent on the yields of the oxidation products (**5** and **6**). The results are summarized in Table 1. As expected, the oxidation rate under O_2_ was higher than that under air (entries 1 and 2). An increase of temperature from room temperature to 50 °C led to the increase of the yields (entry 3). Generally, nonpolar *m*-xylene at 95 °C or higher was the standard reaction condition in the reported AC/O_2_ radical oxidation system^15^,^16^. In our work, various organic solvents were examined and polar solvents, especially acetonitrile, showed more improvement than nonpolar solvents (entries 4–10). In order to optimize the environmental properties and to achieve the minimal toxicity, the oxidation of **4** was carried out in water or under solvent-free conditions. Although the substrate **4** and the oxidation products have relatively low solubility in water, the on-water reaction gave the expected products smoothly (entry 11)^20^. The effect of temperature on these on-water reactions was further investigated, and increasing the reaction temperature from 50 °C to 85 °C led to an increase of the yields of both **5** and **6** (entries 12 and 13).

It should be noted that the pressure of the oxygen inside the reaction tube could also affect the yield. When the mode reaction of **4** was carried under oxygen atmosphere without sealing, the reaction speed was lower than that under the sealed condition and the final yield of **6** was only about 60% at 24 h.

During the reaction optimization, three types of AC, **I**, **II** and **III**, and graphite powder **IV** were used to examine the influence of the surface area and the content of Fe on the yields. As shown in Table 2, **II** and **III** having more surface area than **I**, exhibited higher activity. While **III** contained much lower Fe than **II**, they showed almost the same reactivity (entries 1–3). In contrast, using **IV** with less surface area but more Fe content than the three types AC, no oxidation product was yielded after the reaction (entry 8). Thus, we believe that the surface area of AC plays a more critical role than Fe in the reaction. The yield of the oxidation product was also affected by the amount of AC. The amount of AC **III** was reduced from 40 to 30 mg/mmol without affecting the yields of **5** and **6**, and the yields decreased significantly when 20, 10 or 5 mg/mmol of AC was used (entries 3–7).

**Figure 2:**
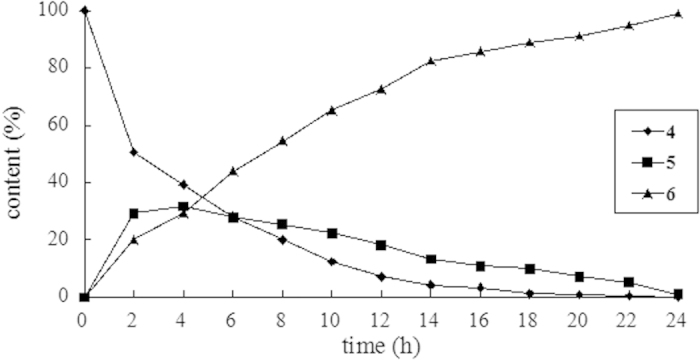
Time course for the substrate **4**, products **5** and **6** using AC **III.** The yields were determined by HPLC analysis.

Figure 2 shows the effect of time on the reaction. Substrate **4** was gradually consumed till undetectable by hour 20. The amount of **5** increased to 31.6% by hour 4 and then slowly decreased to less than 1% by hour 24. On the other hand, product **6** increased consistently from the start and reached at 98.8% at the end. Extending the reaction time to 26 hours did not improve the yield of the reaction.

The observation suggested that **5** was generated during the aerobic oxidation process and it could be converted to the corresponding carbonyl compound (**6**). It is noteworthy that benzylic alcohol (compound **5**) has never been documented before in the oxidation of alkylarenes to the corresponding carbonyl compounds in the AC/O_2_ system. To confirm the oxidized conversion of **5** to **6**, compound **5**, which was prepared *via* reduction of **6** with NaBH_4_ in ethanol, was treated with AC **III** in H_2_O under O_2_ at 85 °C for 12 h, to give **6** in a nearly quantitative yield.

The recycling of AC was subsequently tested in the reaction utilizing of **4** under the aforementioned conditions. In each cycle, AC **III** was separated by filtration and washed with EtOAc, and then used directly in the next cycle without further treatment. The second reaction produced excellent yields (85%) and the third reaction gave a slightly lower yield (81%).

Generally, the mechanism of aerobic oxidation with O_2_ involves a free radical or singlet oxygen^4^,^5^. To evaluate the presence of free radical species in this oxidation, 1.5 equivalents of radical scavenger 2,6-di-tert-butyl-4-methylphenol (BHT) or 2,2,6,6-tetramethyl-1-piperidinyloxy (TEMPO), was added to the reaction mixture in water or water/acetonitrile (1/1, v/v). It was found that the oxidation was unaffected. In addition, the reaction was shown to be unaffected when adding 1.5 equivalents of singlet oxygen quencher (diazabicyclooctane, DABCO) or protecting from the light.

**Figure 3:**
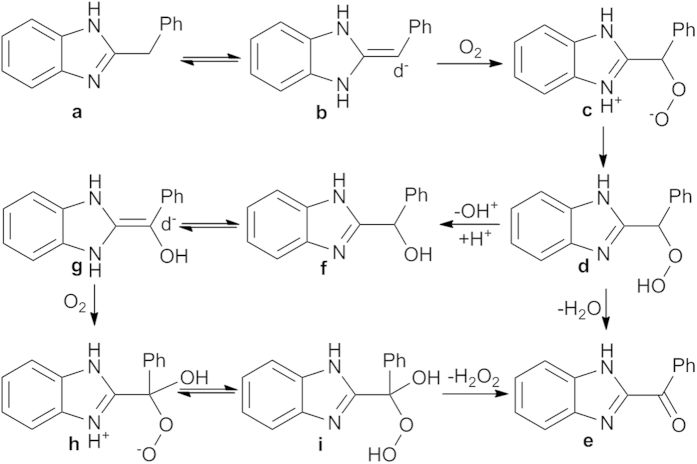
The proposed mechanism of the aerobic benzylic oxidation of 2-benzylbenzo[δ]imidazoles by AC in water.

The detailed mechanism requires further study, however, based on the available data we have and the base-mediated aerobic oxidation involving deprotonation at the carbonyl position described^25^,^26^,^27^, a possible mechanism of the oxidation is proposed in Fig. 3. The starting material (**a**) absorbed on AC could be assumed to exist in form (**b**) with an electric rich centre on the benzyl sp^2^ carbon. The electric density of the benzylic position could be evaluated using computational modeling (Fig. S1). The electrophilic addition of molecular oxygen absorbed on AC to the enamine tautomer **b** formed an intermediate zwitterionic peroxide (**c**), which is subsequently converted to the hydroperoxide (**d**) *via* intra or intermolecular abstraction of a proton. A portion of **d** is dehydrated to give 2-benzoylbenzo[d]imidazole (**e**), while the other **d** is converted into 2-hydroxybenzylbenzo[d]imidazole (**f**). Through a similar process, the secondary benzylic alcohol **f** was oxidized to the corresponding carbonyl compound **e**.

To extend the scope of the procedure and further understand the relationship between the oxidation yield and electric density of the benzylic position, the optimized reaction conditions were applied to a number of 2-benzylbenzimidazoles that are analogues of potential CB2 agonists^28^. As shown in Table 3, 2-benzylbenzo[d]imidazoles bearing electron-withdrawing groups on the benzene ring (entries 4, 8, and 12) generally afforded the higher yield than substrates with electron-donating groups (entries 5, 9 and 13).

Computational electric density of the benzylic position of the substrates was performed with the Jaguar 4.0 software utilizing the B3LYP hybrid density functional method. Table 3 shows that the oxidation yield is closely associated with the benzylic electric density. The substrates carrying three OCH_3_ (entries 5, 9 and 13) generally have lower yields due to the low electric density. For the substrates in entries 3, 7 and 11 that have higher electric density at the benzylic position, the reactions were slightly blocked by the steric hindrance from the chloro group at the ortho position of the benzene ring.

Encouraged by the above results, this method was tested for the synthesis of 2-benzoylbenzo[d]oxazoles that are analogues of fatty acid amide hydrolase inhibitors^29^. Table 4 revealed that 2-benzylbenzo[d]oxazoles are good substrates for the oxidation process. The electronic and steric effects of the substituents are similar with those of 2-benzylbenzo[d]imidazoles. The substrates carrying three OCH_3_ (entries 4 and 13) have low yields due to the low benzylic electric density. In addition, we believe that the relatively lower benzylic electric density of the benzoxazoles caused their lower yields compared with the benzimidazoles in Table 3.

To understand the efficiency of our protocol with respect to others for benzylic oxidation of the 2-benzylbenzimidazoles, the E Factor (cost and environmental impact of the process) and mass intensity of each method are summarized in Table S1 of the Supporting Information. Comparing the oxidants, solvents and yields, it is clear to see that the present conditions are more efficient, clean, and comparatively more sustainable.

In summary, an aerobic benzylic oxidation promoted by commercially available, inexpensive, reusable activated carbon in the green solvent water has been developed. It leads to an environmentally friendly and efficient approach to the synthesis of potential bioactive molecules including 2-benzoylbenzimidazoles and 2-benzoylbenzoxazoles. For the first time, secondary benzylic alcohol (**5**) was found during the activated carbon catalyzed aerobic benzylic oxidation and it could be converted to the corresponding carbonyl compound (**6**) under the standard conditions. The reusable AC maintains a high efficiency including the 3rd run without further treatment. Available evidences indicate that the rate of this oxidation is unaffected by radical scavengers, singlet oxygen quenchers and light irradiation. Preliminary research suggests that the oxidation mechanism may involve the intermediate hydroperoxide and that a portion of the final carbonyl product is obtained through a secondary benzylic alcohol intermediate. In addition, the oxidation yield is closely associated with the benzylic electric density that was calculated by using the Schrödinger software. Efforts to further apply this method to the construction of other potentially valuable organic molecules are currently underway.

## Methods

### Reagents and equipments

Unless otherwise noted, all the materials were obtained from commercially available sources and were used without purification. Thin-layer chromatography was performed on GF254 silica gel plates to monitor the reaction, and the plates were examined under UV light. The purification of the products was performed using column chromatography (60 Å, 200–300 mesh, Qingdao Ocean Chemicals or 120 Å, S-50mm, YMC Co., Ltd.), or silica gel plates (0.25 mm layer, Qingdao Ocean Chemicals) with the designated solvents. IR spectra were obtained using a JASCO FT/IR-480 plus spectrometer. ^1^H and ^13^C NMR spectra were taken in CDCl3 solution on Bruker ARX-300 and Bruker AV-600 spectrometers with TMS as the internal reference. Chemical shifts were reported in ppm downfield from tetramethylsilane and proton–proton coupling constants (*J*) in Hz. ESI-MS spectrawere performed on a Finigan LCQ Advantage MAX mass spectrometer.

### General experimental section

A mixture of substrate (2 mmol) and activated carbon III (60 mg) in water (5 mL) was placed in a sealed tube (100 mL). The system was purged with molecular oxygen and sealed. The reaction mixture was heated to 85 °C and stirred for 24 h. Upon completion, the reaction mixture was cooled to room temperature and filtered. After the filtrate was concentrated, the product was isolated by silica gel column chromatography and analyzed by MS, ^1^H-NMR and ^13^C-NMR. See the supporting information for the characterization details.

## Additional Information

**How to cite this article**: Bao, K. *et al*. Activated carbon for aerobic oxidation: Benign approach toward 2-benzoylbenzimidazoles and 2-benzoylbenzoxazoles synthesis. *Sci. Rep.* **5**, 10360; doi: 10.1038/srep10360 (2015).

## Supplementary Material

Supplementary Information

## Figures and Tables

**Table 1:** Optimization of benzylic oxidation of 4^a^.

**Entry**	**Solvent**	**Atm.**	**Temp (°C)**	**Yield^b^ 5**	**(%) 6**
1	EtOH	air	r. t.	5.2^c^	5.6^c^
2	EtOH		r. t.	7.9^c^	6.3^c^
3	EtOH		50	8.3	5.9
4	MeOH		50	11.1	1.4
5	Acetonitrile		50	12.0	6.1
6	Acetone		50	7.1	8.2
7	EtOAc		50	11.2	7.9
8	THF	O_2_	50	5.4	5.5
9	Toluene		50	<1	5.3
10	*m*-Xylene		50	<1	3.4
11	H_2_O		50	6.7	4.2
12	H_2_O		70	11.4	8.4
13	H_2_O		85	31.6	29.5
14	solvent-free		85	8.5	12.7

^a^Reaction conditions: **4** (2 mmol), AC **III** (Aladdin Inc., 60 mg) and solvent (5 mL).

^b^Yields were based on HLPC analysis.

^c^Isolated yields for 12 h.

**Table 2:** Type and amount effects of AC and graphite powder^a^.

**Entry**	**Type**	**Surface area (m**^**2**^**/g)**	**Fe (ppm)**	**Amount (mg/mmol of 4)**	Yield^b^ 5	**(%) 6**
1	**I**	600	2×10^5^	30	6.2	4.5
2	**II**	1100	5×10^5^	30	22.1	32.5
3	**III**	1100	0.02	30	31.6	29.5
4	**III**	1100	0.02	40	28.2	32.1
5	**III**	1100	0.02	20	26.4	25.0
6	**III**	1100	0.02	10	9.8	21.5
7	**III**	1100	0.02	5	5.9	10.1
8	**IV**	<50	5×10^6^	30	< 1	< 1

^a^Substrate **4** (2 mmol), AC (60 mg) and H_2_O (5 mL), 85 °C, 4 h.

^b^Yields were based on HLPC analysis.

**Table 3:**
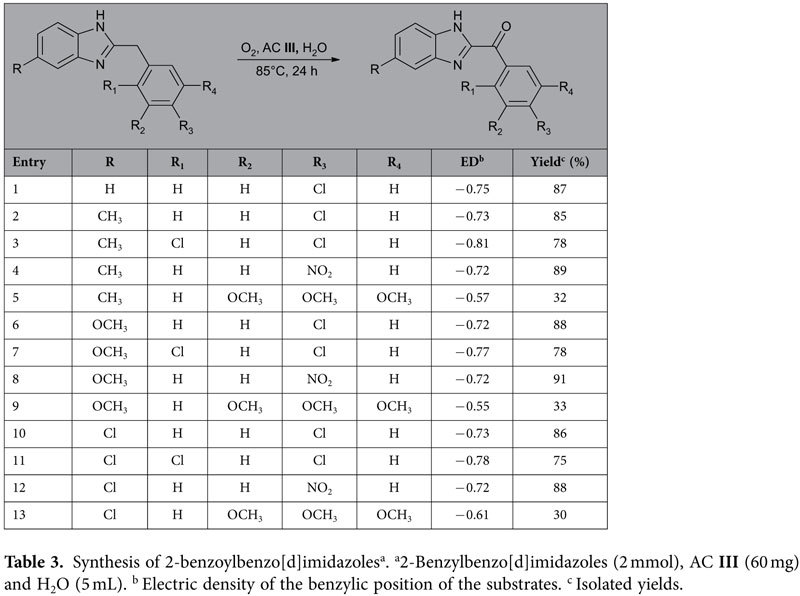
Synthesis of 2-benzoylbenzo[d]imidazoles^a^.

**Table 4:**
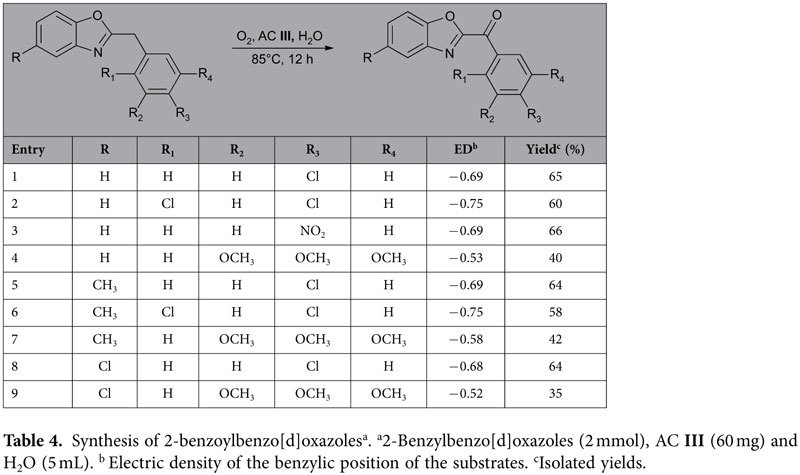
Synthesis of 2-benzoylbenzo[d]oxazoles^a^.
