# Supplementary material for: Activated carbon for aerobic oxidation: Benign approach toward 2-benzoylbenzimidazoles and 2-benzoylbenzoxazoles synthesis
Source: Sci Rep. 2015 Jun 4;5:10360. doi: 10.1038/srep10360 (PMC4603700; doi:10.1038/srep10360)

Supplementary Information

Activated carbon for aerobic oxidation: Benign approach toward 2-benzoylbenzimidazoles and 2-benzoylbenzoxazoles synthesis

Kai Bao[1,2], Fuqing Li[1], Hanjing Liu[1], Zhiwei Wang[1], Qirong Shen[1], Jian Wang[1], and Weige Zhang [1*]

[1]Key Laboratory of Structure-Based Drug Design & Discovery, Ministry of Education

Shenyang Pharmaceutical University, 103 Wenhua Road, Shenyang 110016 (China)

[2]Division of Hematology/Oncology, Department of Medicine, Beth Israel Deaconess Medical Center,Harvard Medical School, Boston, MA 02215 (USA)

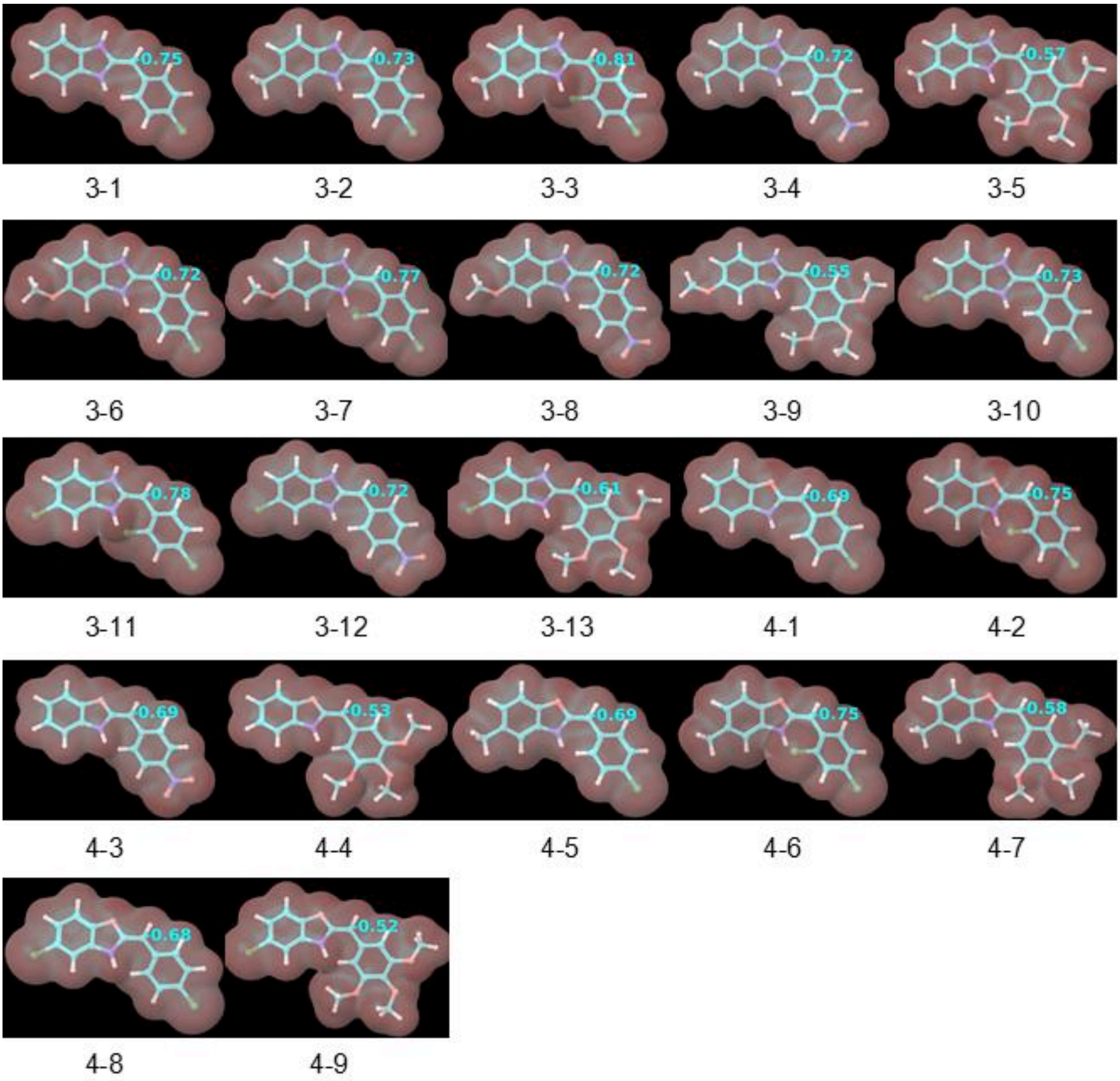

Figure S1: Electric density of the substrates in Table 3 and 4 in the form of the enamine tautomer.

Method: Calculations of the electric density were carried out with the Jaguar 4.0 software within Schrodinger (2014.1) using the B3LYP hybrid density functional method. Geometries were optimized using the 6-31G (d) basis set, and single point energies were calculated using the 6-311+G (d,p) basis set.

Table S1. Comparison of reported procedures for the benzylic oxidation of 2-benzylbenzimidazoles

a.

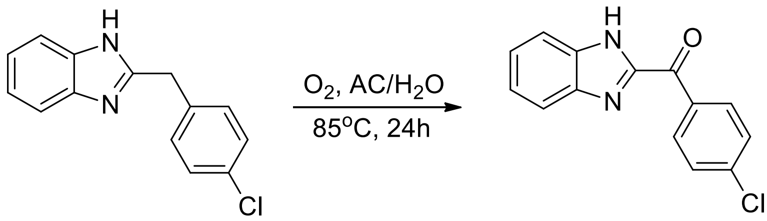

Raw Materials Used		Product and Waste	
Compound **4**	485.40 mg	Compound **6**	446.64 mg
AC **III**	60.00 mg	Aqueous waste	5.00 g
Water	5.00 g (5 mL)		
Total	5.54 g	Total	5.45 g

E Factor (E) = (5 g of waste produced / 446.64 mg of product) = 11.19
Mass Intensity = (5.54 g of raw material used / 446.64 mg of product) = 12.40

b.

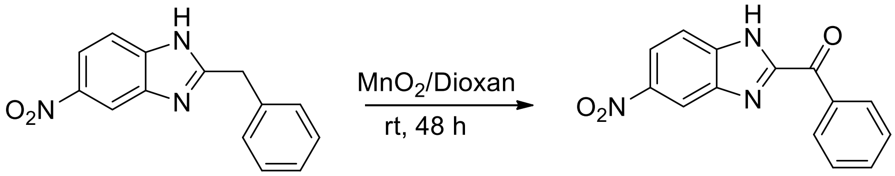

H. C. Ooi and H. Suschitzky, *J. Chem. Soc., Perkin Trans. 1*, 1982, 2871.

Raw Materials Used		Product and Waste	
Compound **5**	0.20 g	Compound **4**	0.16 g
MnO_2	1.00 g	Solid waste	1.00 g
Dioxan	10.30 g (10 mL)	Organic waste	10.30 g
Total	11.50 g	Total	11.46 g

E Factor (E) = (11.46 g of waste produced / 0.16 g of product) = 71.63
Mass Intensity = (11.50 g of raw material used / 0.16 g of product) = 71.88

c.

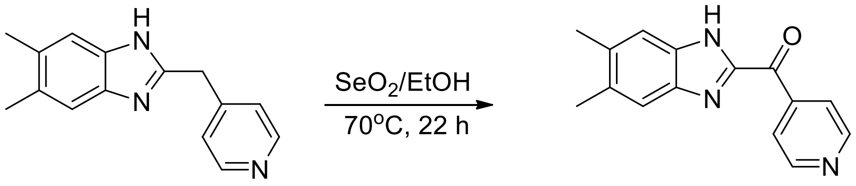

E. Alcalde, M. Gisbert, J. M. Pons and L. Perez-Garcia, *Tetrahedron*, 1996, 52, 15197.

Raw Materials Used		Product and Waste	
Compound **8b**	3.30 g	Compound **11b**	1.32 g
SeO_2	1.76 g	Organic waste	60.94 g
EtOH	59.18 g (75 mL)		
Total	64.24 g	Total	62.26 g

E Factor (E) = (60.94 g of waste produced / 1.32 g of product) = 46.17
Mass Intensity = (64.24 g of raw material used / 1.32 g of product) = 48.67

d.

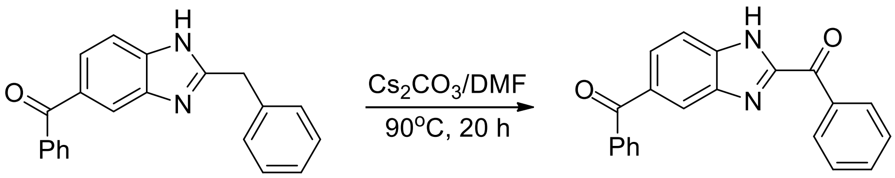

A. D. Santos, L. E. Kaïm and L. Grimaud, *Org. Biomol. Chem.*, 2013, 11, 3282.

Raw Materials Used		Product and Waste	
Compound **3d**	62.47 mg	Compound **4d**	47.65 mg
Cs_2CO_3	65.16 mg	Organic waste	1.00 g
DMF	0.94 g (1 mL)		
Total	1.07 g	Total	1.05 g

E Factor (E) = (1.00 g of waste produced / 47.65 mg of product) = 20.99
Mass Intensity = (1.07 g of raw material used / 47.65 mg of product) = 22.46

Note:
1. >98 % catalyst recovered in our method.
2. Calculation did not consider for solvent used for column chromatography.

2-Benzoylbenzimidazoles

2-(4-Chloro)benzoylbenzimidazole (**6**, Table 3, entry 1)

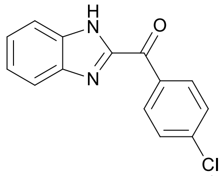

M. p. 226-228°C; ^{1}H-NMR (600MHz, DMSO-d_6): □=13.54 (s, 1H), 8.61 (d, J=8.58 Hz, 2H), 7.87 (s, 1H), 7.66 (d, J=8.58 Hz, 2H), 7.60 (s, 1H), 7.40 (s, 1H), 7.32 (s, 1H); ^{13}C-NMR (DMSO-d_6): □=182.6, 148.2, 143.6, 139.2, 134.7, 133.2, 129.1, 126.4, 123.8, 121.8, 113.3; MS: *m/z* (%): 257.0 ($[M+H]^+$).

2-(4-Chlorohydroxybenzyl)-benzo[d]imidazole (**5**)

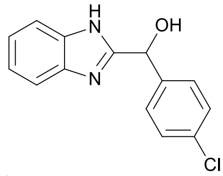

^{1}H-NMR (600MHz, DMSO-d_6): □=7.41 (m, 2H), 7.23 (d, J=7.15 Hz, 2H), 7.18 (m, 2H), 7.13 (d, J=7.15 Hz, 2H); MS: *m/z* (%): 259.0 ($[M+H]^+$).

2-(4-Chloro)benzoyl-5-methylbenzimidazole (Table 3, entry 2)

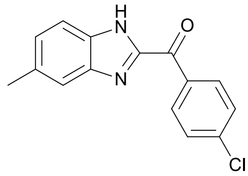

M. p. 200-203°C; ^{1}H-NMR (300MHz, DMSO-d_6): □=10.54 (s, 1H), 8.66 (d, J=8.60 Hz, 2H), 7.82 (d, J=8.45 Hz, 1H), 7.53 (d, J=8.60 Hz, 2H), 7.20 (d, J=8.45 Hz, 1H), 2.52 (s, 3H); ^{13}C-NMR (DMSO-d_6): □=182.4, 147.9, 144.1, 142.0, 139.1, 136.2, 134.7, 133.1, 129.0, 125.7, 121.4, 112.6, 22.0; MS: *m/z* (%): 268.9 ($[M-H]^-$).

2-(2,4-Dichloro)benzoyl-5-methylbenzimidazole (Table 3, entry 3)

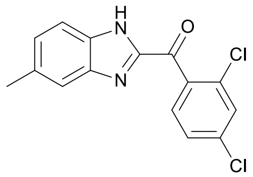

M. p. 223-225°C; ^{1}H-NMR (300MHz, DMSO-d_6): □=13.61 (s, 1H), 7.84 (m, 2H), 7.63 (m, 2H), 7.46 (m, 1H), 7.19 (m, 1H), 2.44 (s, 3H); ^{13}C-NMR (DMSO-d_6): □=185.3, 147.7, 136.5, 136.2, 136.1, 133.2, 132.5, 132.4, 129.9, 129.0, 127.6, 126.4, 121.2, 112.9, 21.9; MS: *m/z* (%): 305.0 ($[M+H]^+$), 327.0 ($[M+Na]^+$).

2-(4-Nitrol)benzoyl-5-methylbenzimidazole (Table 3, entry 4)

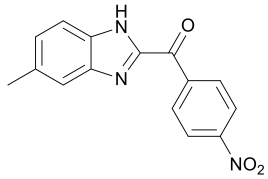

M. p. 212-214°C; ^{1}H-NMR (300MHz, DMSO-d_6): □=13.53 (s, 1H), 8.72 (d, J=8.60 Hz, 2H), 8.41 (d, J=8.60 Hz, 2H), 7.19 -7.70(m, 3H), 2.45 (s, 3H); ^{13}C-NMR (DMSO-d_6): □=182.4, 150.3, 147.6, 144.1, 142.0, 141.0, 136.6, 135.1, 132.5, 125.9, 123.7, 121.5, 112.7, 22.0; MS: *m/z* (%): 279.9 ($[M-H]^-$).

2-(3,4,5-Trimethoxy)benzoyl-5-methylbenzimidazole (Table 3, entry 5)

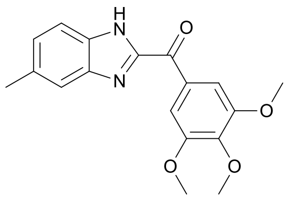

M. p. 195-197°C; ^{1}H-NMR (300MHz, DMSO-d_6): □=13.33 (s, 1H), 8.05 (s, 2H), 7.73 (d, J=8.45 Hz, 2H), 7.39 (s, 1H), 7.15 (dd, J=8.45 Hz, J=1.12 Hz), 3.89 (s, 6H), 3.81 (s, 3H)), 2.46 (s, 3H); ^{13}C-NMR (DMSO-d_6): □=182.1, 152.9, 148.2, 143.1, 134.8, 131.9, 129.1, 125.6, 121.4, 112.5, 109.3, 60.7, 56.6, 19.1; MS: *m/z* (%): 327.1 ($[M+H]^+$), 349.1 ($[M+Na]^+$).

2-(4-Chloro)benzoyl-5-methoxybenzimidazole (Table 3, entry 6)

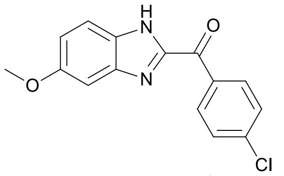

M. p. 169-171°C; ^{1}H-NMR (300MHz, DMSO-d_6): □=13.40 (s, 1H), 8.58 (d, J=7.05 Hz, 2H), 7.65 (m, 3H), 6.98 (m, 1H), 3.8 (s, 3H); ^{13}C-NMR (DMSO-d_6): □=182.0, 159.3, 150.3, 147.3, 141.2, 138.6, 136.0, 132.5, 123.8, 122.8, 115.6, 94.2, 60.0; MS: *m/z* (%): 284.9 ($[M-H]^-$).

2-(2,4-Dichloro)benzoyl-5-methoxybenzimidazole (Table 3, entry 7)

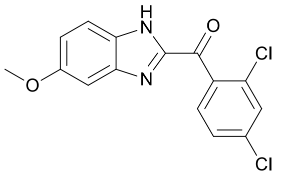

M. p. 202-203°C; ^{1}H-NMR (300MHz, DMSO-d_6): □=13.56 (s, 1H), 7.84 (d, J=8.30, 1H), 7.79 (d, J=1.91, 1H), 7.62 (m, 2H), 7.02 (m, 2H), 3.82 (s, 3H); ^{13}C-NMR (DMSO-d_6): □=184.6, 159.4, 147.3, 138.6, 136.3, 132.3, 131.9, 129.8, 129.1, 127.5, 122.9, 115.6, 94.2, 56.0; MS: *m/z* (%): 318.9 ($[M-H]^-$).

2-(4-Nitrol)benzoyl-5-methoxybenzimidazole (Table 3, entry 8)

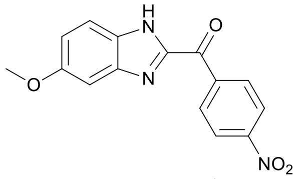

M. p. 216-218°C ; ^{1}H-NMR (300MHz, DMSO-d_6): □=13.52 (s, 1H), 8.70 (d, J=8.67 Hz, 2H), 8.41 (d, J=8.67 Hz, 2H), 7.70 (s, 1H), 7.00 (m, 2H), 3.83 (s, 3H); ^{13}C-NMR (DMSO-d_6): □=181.8, 159.0, 147.6, 138.9, 138.5, 135.7, 134.8, 133.0, 128.9, 122.6, 115.2, 94.3, 55.9; MS: *m/z* (%): 295.9 ($[M-H]^-$).

2-(3,4,5-Trimethoxy)benzoyl-5-methoxybenzimidazole (Table 3, entry 9)

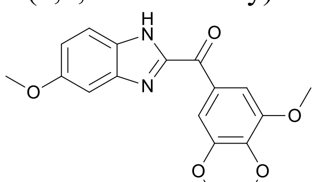

M. p. 150-153°C; ^{1}H-NMR (300MHz, DMSO-d_6): □=13.32 (s, 1H), 8.03 (s, 2H), 7.73 (d, J=8.81 Hz, 1H), 7.00 (s, 1H), 6.95 (d, J=8.81 Hz, 1H), 3.88 (s, 9H), 3.83 (s, 3H); ^{13}C-NMR (DMSO-d_6): □=181.6, 167.4, 158.8, 152.9, 142.9, 138.4, 135.5, 131.1, 129.1, 122.6, 114.9, 109.3, 94.3, 60.7, 56.5, 55.9; MS: *m/z* (%): 343.1 ($[M+H]^+$), 365.0 ($[M+Na]^+$).

2-(4-Chloro)benzoyl-5-chlorobenzimidazole (Table 3, entry 10)

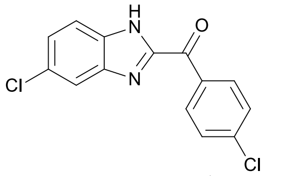

M. p. 224-226°C; ^{1}H-NMR (300MHz, DMSO-d_6): □=8.57 (d, J=8.52 Hz, 2H), 7.77 (s, 1H), 7.74 (d, J=8.81 Hz, 1H), 7.66 (d, J=8.52 Hz, 2H), 7.37 (dd, J=8.81 Hz, J=1.69 Hz, 1H); ^{13}C-NMR (DMSO-d_6): □=182.5, 149.2, 142.3, 139.4, 135.2, 134.4, 133.2, 130.7, 129.1, 124.4, 120.9, 112.9; MS: *m/z* (%): 288.8 ($[M-H]^-$).

2-(2,4-Dichloro)benzoyl-5-chlorobenzimidazole (Table 3, entry 11)

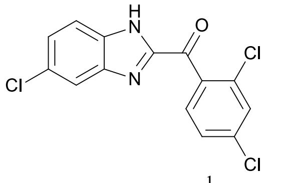

M. p. 182-184°C; ^{1}H-NMR (300MHz, DMSO-d_6): □=13.76 (s, 1H), 7.78-7.90 (m, 2H), 7.63 (m, 3H), 7.40 (s, 1H); ^{13}C-NMR (DMSO-d_6): □=185.0, 149.0, 144.3, 136.9, 135.6, 132.7, 132.5, 131.2, 130.0, 128.3, 127.5, 124.5, 121.2, 112.9; MS: *m/z* (%): 322.8 ($[M-H]^-$)

2-(4-Nitrol)benzoyl-5-chlorobenzimidazole (Table 3, entry 12)

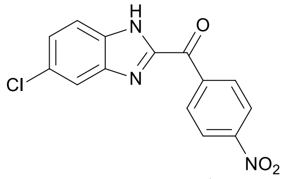

M. p. 234-235°C; ^{1}H-NMR (300MHz, DMSO-d_6): □=13.61 (s, 1H), 8.68 (d, J=8.52 Hz, 2H), 8.37 (d, J=8.52 Hz, 2H), 7.75 (s, 2H), 7.36 (m, 1H); ^{13}C-NMR (DMSO-d_6): □=181.9, 150.2, 148.6, 140.3, 132.4, 131.8, 130.5, 129.0, 124.4, 120.7, 114.6, 111.1; MS: *m/z* (%): 299.8 ($[M-H]^-$)

2-(3,4,5-Trimethoxy)benzoyl-5-chlorobenzimidazole (Table 3, entry 13)

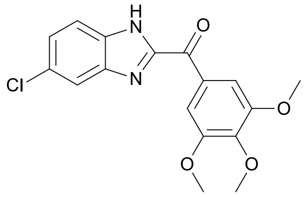

M. p. 201-203°C; ^{1}H-NMR (300MHz, DMSO-d_6): □=13.64 (s, 1H), 8.01 (s, 2H), 7.39-7.83 (m, 3H), 3.89 (s, 6H), 3.81 (s, 3H); ^{13}C-NMR (DMSO-d_6): □=180.0, 152.9, 149.6, 143.3, 132.2, 131.9, 130.7, 129.1, 125.4, 118.3, 109.3, 60.8, 56.6; MS: *m/z* (%): 347.1 ($[M+H]^+$), 369.0 ($[M+Na]^+$).

2-Benzoylbenzoxazoles

2-(4-Chloro)benzoylbenzoxazole (Table 4, entry 1)

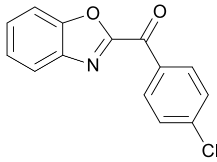

M.p. 106-108°C; ^{1}H-NMR (300MHz, $CDCl_3$): □=8.57 (d, J=8.63 Hz, 2H), 7.94 (d, J=7.79 Hz, 1H), 7.70 (d, J=7.79, 1H), 7.45-7.58 (m, 4H); 13C-NMR ($CDCl_3$): □=179.1, 156.9, 150.4, 141.1, 140.7, 133.30, 132.5, 128.9, 128.6, 125.9, 122.4, 111.9; MS: m/z (%): 257.9 ([M+H]+), 279.9 ([M+Na]+)

2-(2,4-Dichloro)benzoylbenzoxazole (Table 4, entry 2)

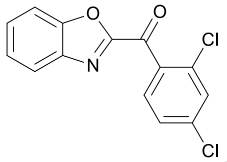

M.p. 102-103°C; ^{1}H-NMR (300MHz, $CDCl_3$): □=7.90 (d, J=8.08, 1H), 7.78 (d, J=8.23, 1H), 7.70 (d, J=8.08 Hz, 1H), 7.59 (dd, J=7.41 Hz, J=1.22 Hz, 1H), 7.54 (dd, J=7.41 Hz, J=1.22 Hz, 1H), 7.49 (dd, J=8.23 Hz, J=1.79 Hz,1H), 7.43 (m, 1H); ^{13}C-NMR ($CDCl_3$): □=180.9, 157.0, 150.9, 140.8, 138.8, 134.0, 133.9, 131.9, 130.7, 129.0, 127.2, 126.1, 122.7, 112.0; MS: *m/z* (%): 291.9 ($[M+H]^+$), 313.9 ($[M+Na]^+$).

2-(4-Nitro)benzoylbenzoxazole (Table 4, entry 3)

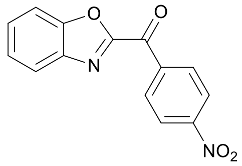

M.p. 106-108°C; ^{1}H-NMR (300MHz, $CDCl_3$): □=8.78 (d, J=9.07 Hz, 2H), 8.41 (d, J=9.07 Hz, 2H), 7.98 (m, 1H), 7.73 (m, 1H), 7.61 (m, 1H), 7.52 (m, 1H); ^{13}C-NMR ($CDCl_3$): □=178.7, 156.5, 150.8, 150.5, 140.6, 139.5, 132.1, 129.2, 126.2, 123.6, 122.7, 112.0; m/z (%): 269.1 ($[M+H]^+$).

2-(3,4,5-Trimethoxy)benzoylbenzoxazole (Table 4, entry 4)

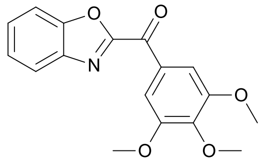

M.p. 89-91°C; ^{1}H-NMR (300MHz, $CDCl_3$): □=7.96 (s, 2H), 7.94 (d, J=8.14 Hz, 1H), 7.73 (d, J=8.14 Hz, 1H), 7.53 (m, 2H), 4.00 (d, 9H); ^{13}C-NMR ($CDCl_3$): □=178.9, 157.3, 152.9, 150.4, 144.0, 140.8, 129.9, 128.4, 125.7, 122.3, 111.9, 108.8, 61.1, 56.4; MS: *m/z* (%): 314.1 ($[M+H]^+$), 336.0 ($[M+Na]^+$).

2-(4-Chloro)benzoyl-5-methylbenzoxazole (Table 4, entry 5)

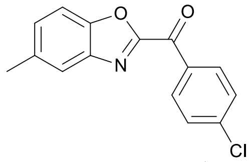

M.p. 107-108°C; ^{1}H-NMR (300MHz, $CDCl_3$): □=8.54 (d, J=8.88 Hz, 2H), 7.70 (m , 1H), 7.57 (d, J=8.47 Hz, 1H), 7.52 (d, J=8.88 Hz, 2H), 7.36 (dd, J=8.47 Hz, J=1.30 Hz, 1H), 2.51 (s, 3H); ^{13}C-NMR ($CDCl_3$): □=179.1, 157.0, 148.7, 141.0, 140.9, 135.9, 133.4, 132.4, 130.1, 128.9, 121.9, 111.2, 21.6; MS: *m/z* (%): 272.0 ($[M+H]^+$).

2-(2,4-Dichloro)benzoyl-5-methylbenzoxazole (Table 4, entry 6)

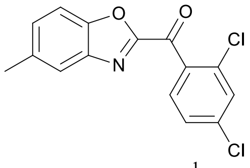

M.p.105-107°C; ^{1}H-NMR (300MHz, $CDCl_3$): □=7.76 (d, J=8.37 Hz, 1H), 7.66 (s, 1H), 7.55 (d, J=8.52 Hz, 1H), 7.53 (d, J=1.87 Hz, 1H), 7.34 (dd, J=8.37 Hz, J=1.87 Hz, 1H), 7.36(m, 1H), 2.50(s, 3H); ^{13}C-NMR ($CDCl_3$): □=180.9, 157.2, 149.2, 141.0, 138.7, 136.2, 134.1, 133.9, 131.9, 130.7, 130.5, 127.2, 122.1, 111.3, 21.6; MS: *m/z*(%): 306.0 ($[M+H]^+$), 338.0 ($[M+Na]^+$).

2-(3,4,5-Trimethoxy)benzoyl-5-methylbenzoxazole (Table 4, entry 7)

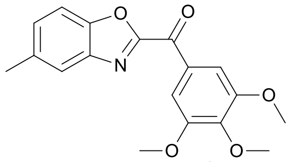

M. p. 100-102°C; ^{1}H-NMR (300MHz, $CDCl_3$): □=7.94 (s, 2H), 7.72 (s, 1H), 7.58 (d, J=8.55 Hz, 1H), 7.37 (dd, J=8.55 Hz, J=1.03, 1H), 3.99 (m, 9H), 2.52 (s, 3H); ^{13}C-NMR ($CDCl_3$): □=179.0, 157.4, 152.9, 148.7, 143.9, 141.0, 135.8, 130.0, 129.8, 121.9, 111.2, 108.8, 61.1, 56.3, 21.5; MS: *m/z* (%): 328.1 ($[M+H]^+$), 350.0 ($[M+Na]^+$).

2-(4-Chloro)benzoyl-5-chlorobenzoxazole (Table 4, entry 8)

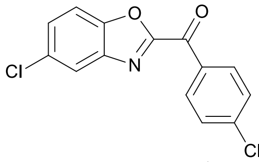

M. p. 112-114°C; ^{1}H-NMR (300MHz, $CDCl_3$): □=8.50 (d, J=8.63 Hz, 2H), 7.90 (s, 1H), 7.61 (d, J=8.71, 1H), 7.51(m, 3H); ^{13}C-NMR ($CDCl_3$): □=178.7, 157.8, 148.9, 141.6, 141.4, 133.0, 132.5, 131.5, 129.1, 129.0, 122.1, 112.7; MS: *m/z* (%): 292.1 ($[M+H]^+$), 313.9 ($[M+Na]^+$).

2-(3,4,5-Trimethoxy)benzoyl-5-chlorobenzoxazole (Table 4, entry 9)

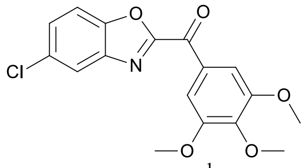

M. p. 107-108°C; ^{1}H-NMR (300MHz, $CDCl_3$): □=7.94 (m , 3H), 7.66 (d, J=8.88 Hz, 1H), 7.53 (dd, J=8.88 Hz, J=1.95 Hz, 1H), 4.00 (m, 9H); ^{13}C-NMR ($CDCl_3$): □=178.5, 158.2, 153.0, 148.9, 144.3, 141.7, 131.3, 129.5, 128.8, 122.1, 112.7, 108.8, 61.1, 56.4; MS: *m/z* (%): 348.0 ($[M+H]^+$), 370.0 ($[M+Na]^+$).

2-(4-Chloro)benzoylbenzimidazole (**6**)

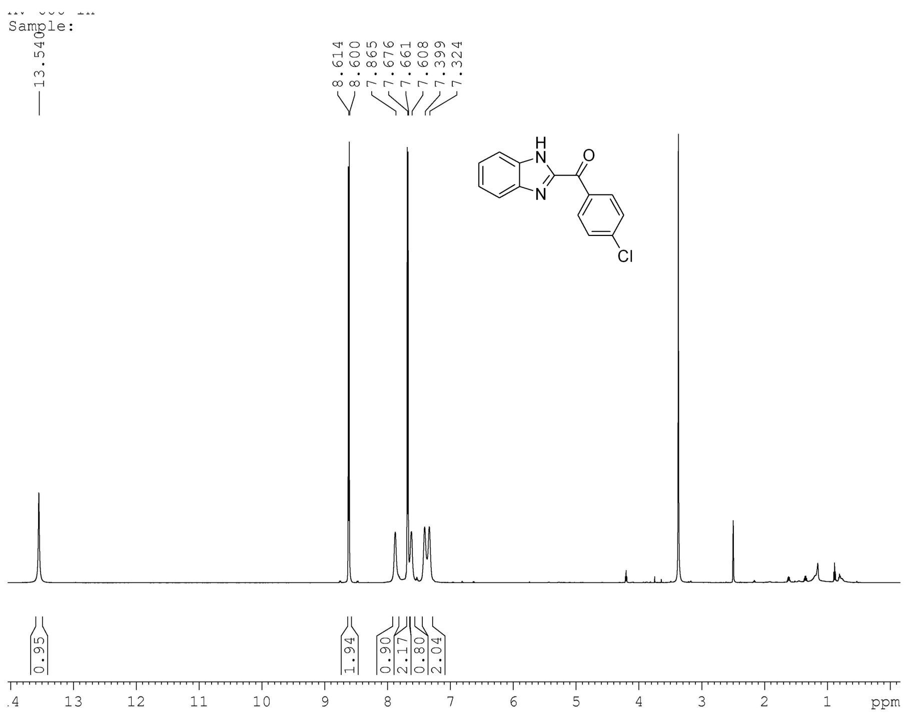

2-(4-Chloro)benzoylbenzimidazole (**6**)

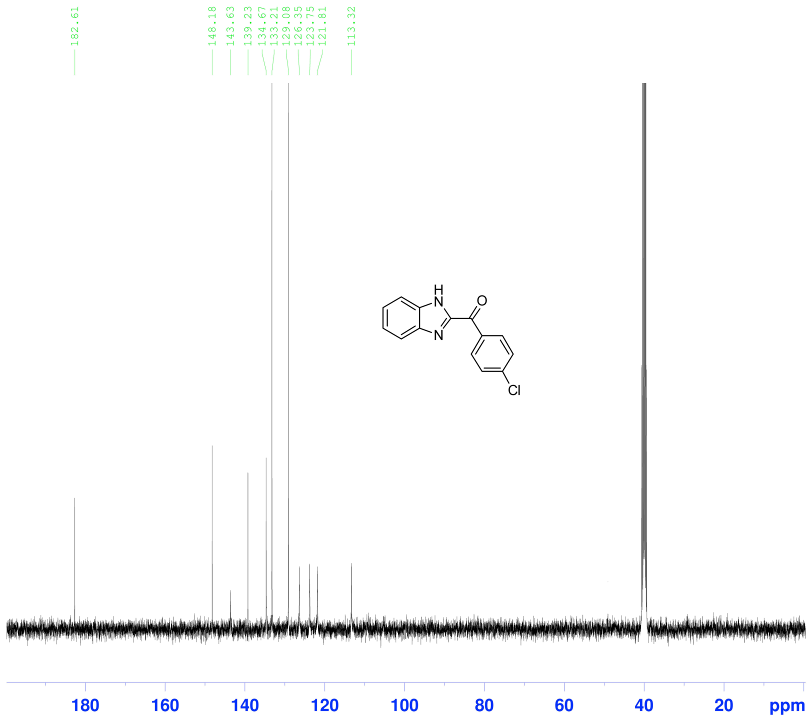

2-(4-Chlorohydroxybenzyl)-benzo[d]imidazole(**5**)

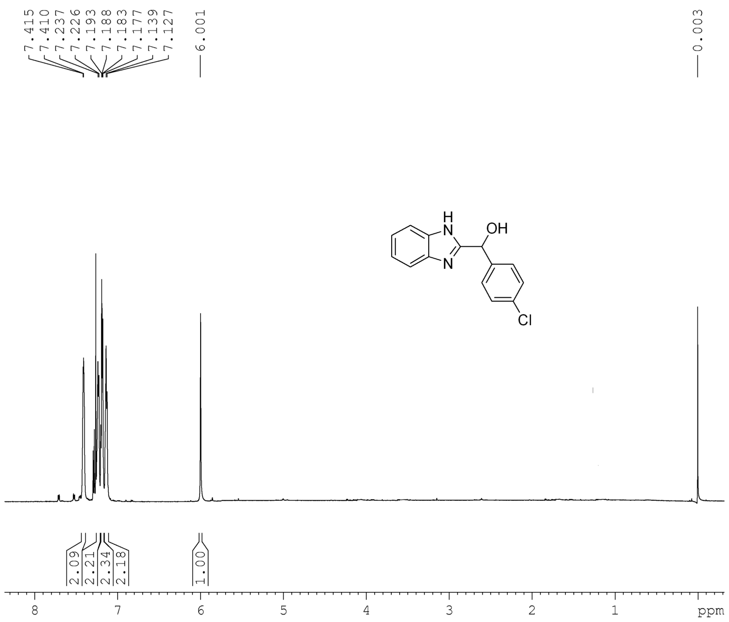

2-(4-Chloro)benzoyl-5-methylbenzimidazole

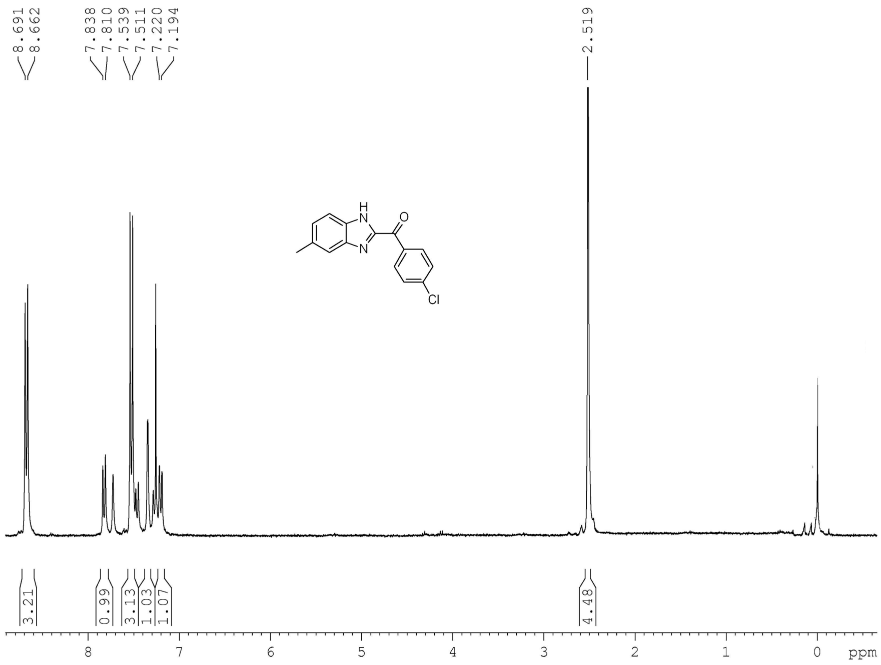

2-(4-Chloro)benzoyl-5-methylbenzimidazole

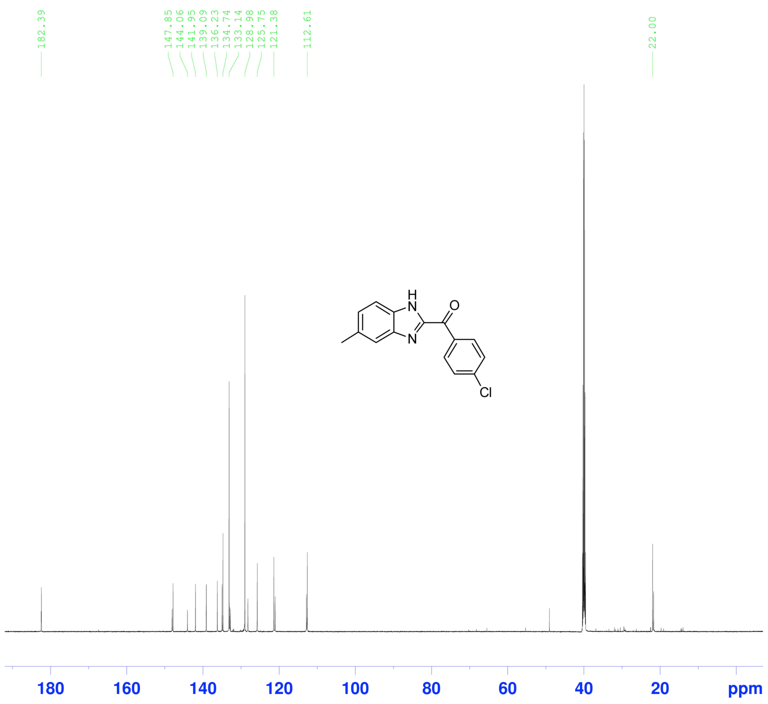

2-(2,4-Dichloro)benzoyl-5-methylbenzimidazole

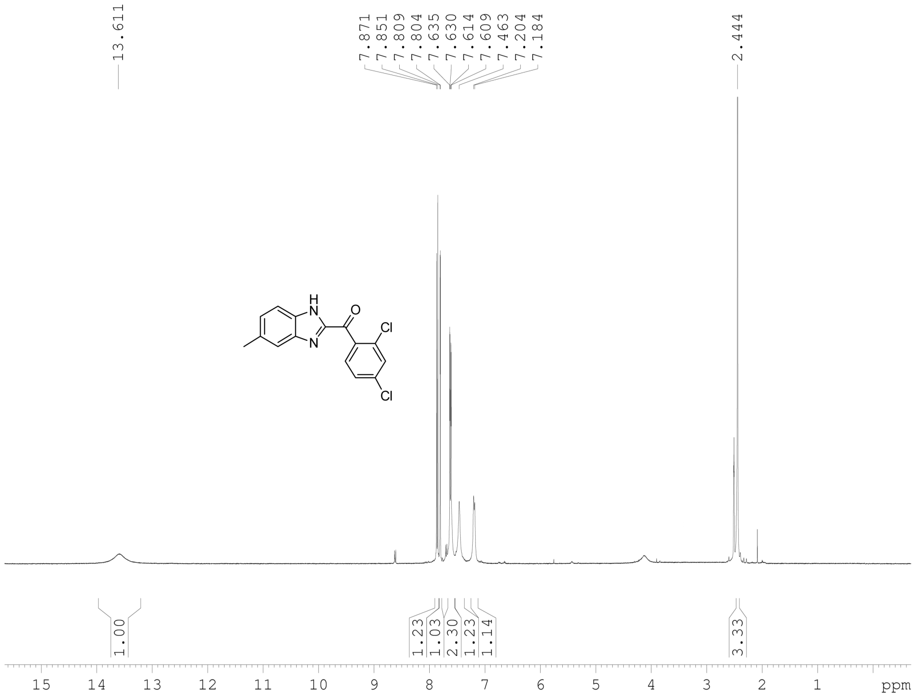

2-(2,4-Dichloro)benzoyl-5-methylbenzimidazole

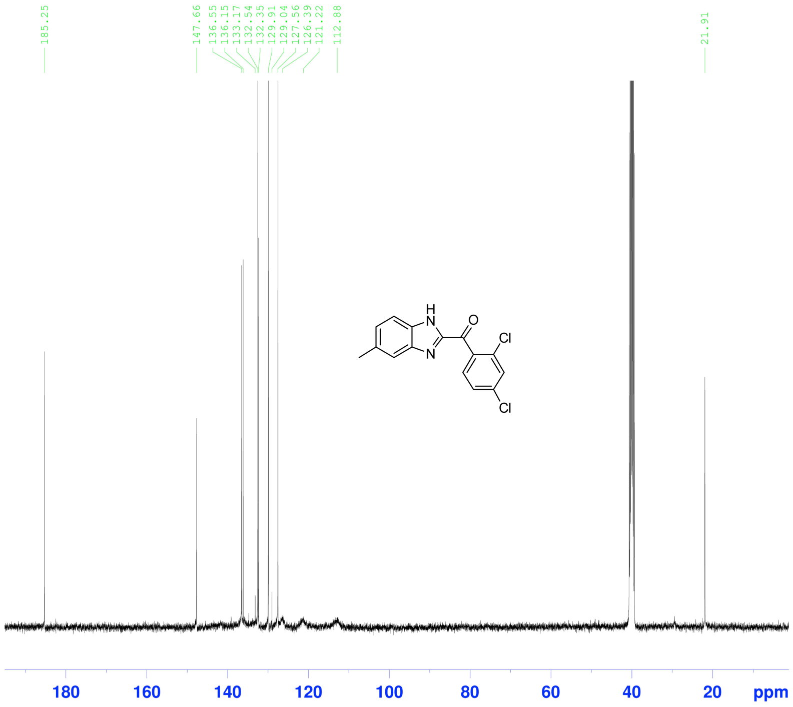

2-(4-Nitrol)benzoyl-5-methylbenzimidazole

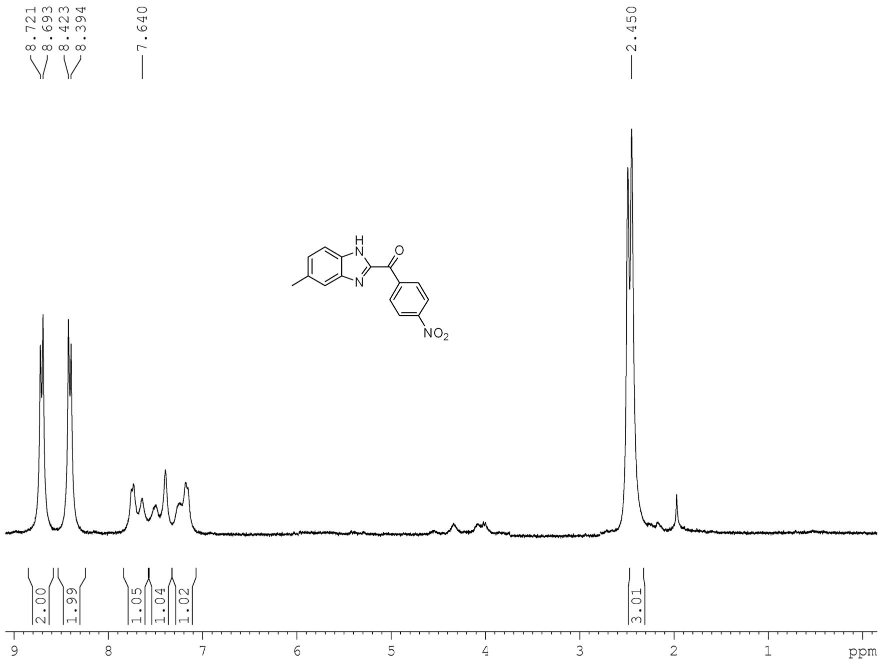

2-(4-Nitrol)benzoyl-5-methylbenzimidazole

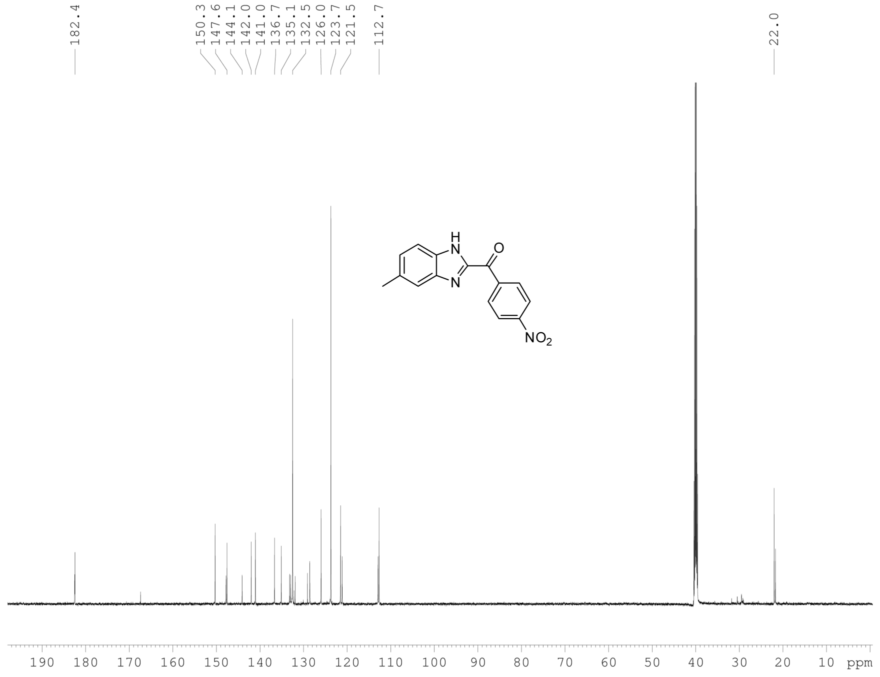

2-(3,4,5-Trimethoxy)benzoyl-5-methylbenzimidazole

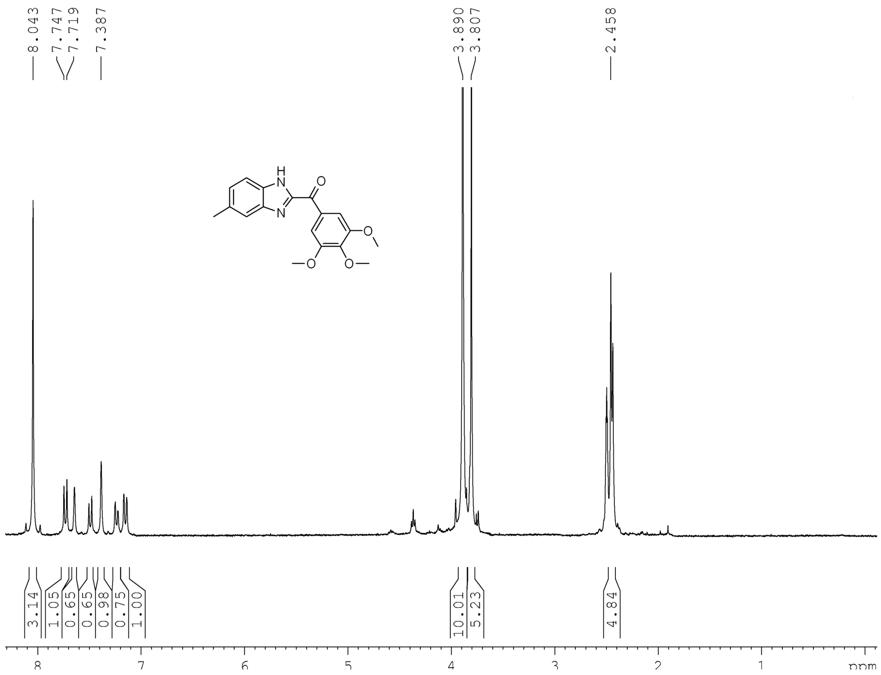

2-(3,4,5-Trimethoxy)benzoyl-5-methylbenzimidazole

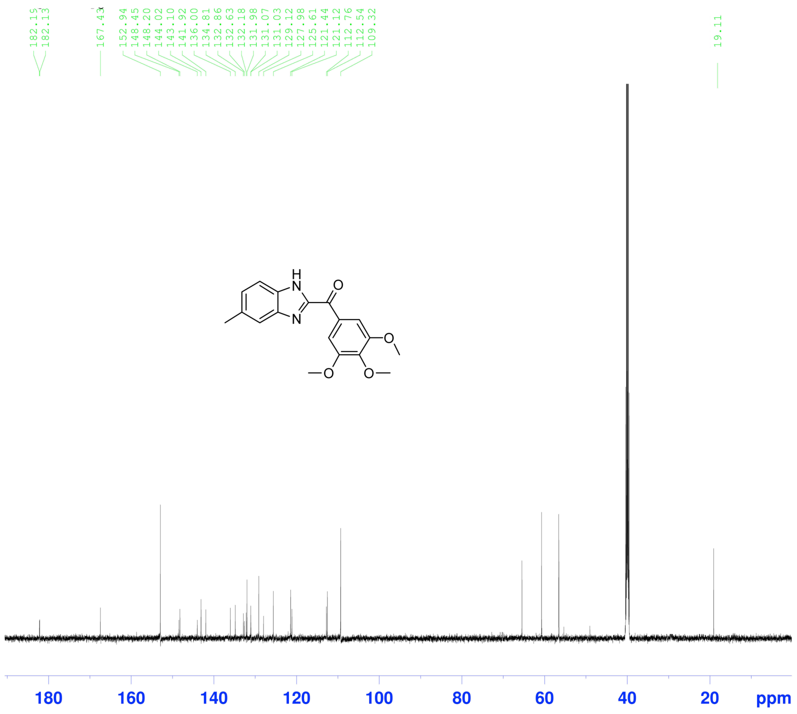

2-(4-Chloro)benzoyl-5-methoxybenzimidazole

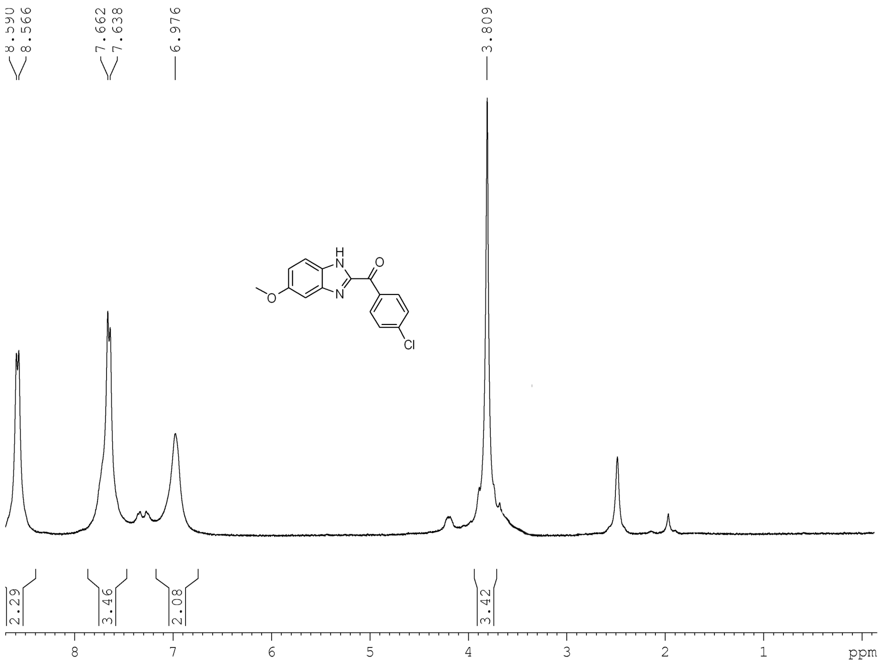

2-(4-Chloro)benzoyl-5-methoxybenzimidazole

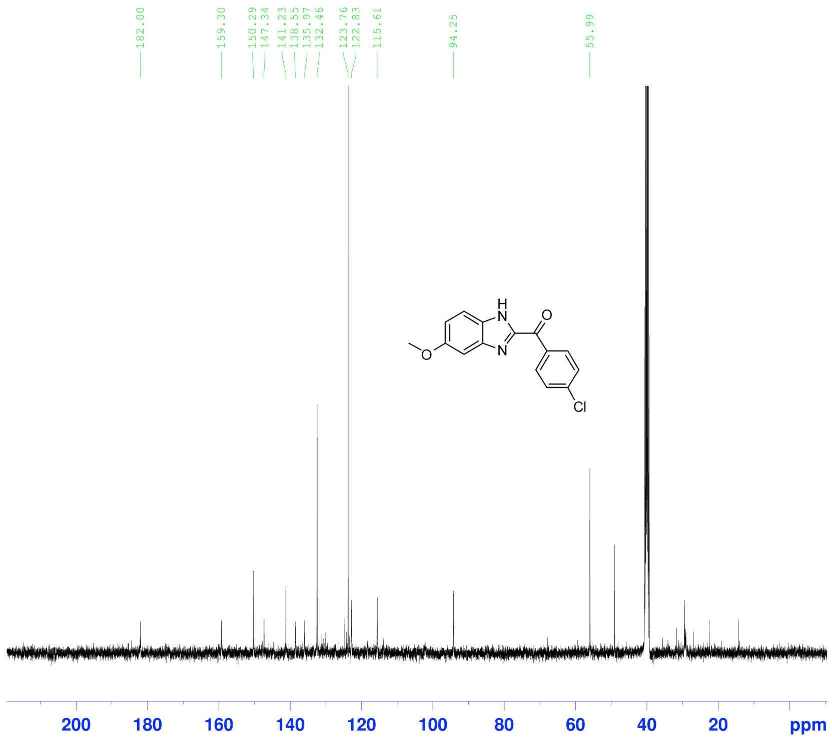

2-(2,4-Dichloro)benzoyl-5-methoxybenzimidazole

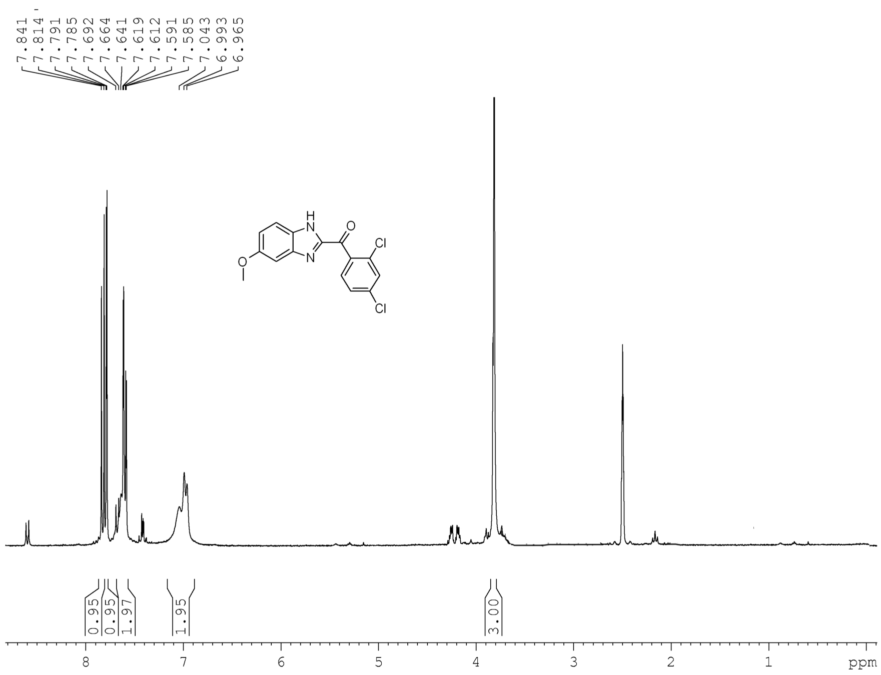

2-(2,4-Dichloro)benzoyl-5-methoxybenzimidazole

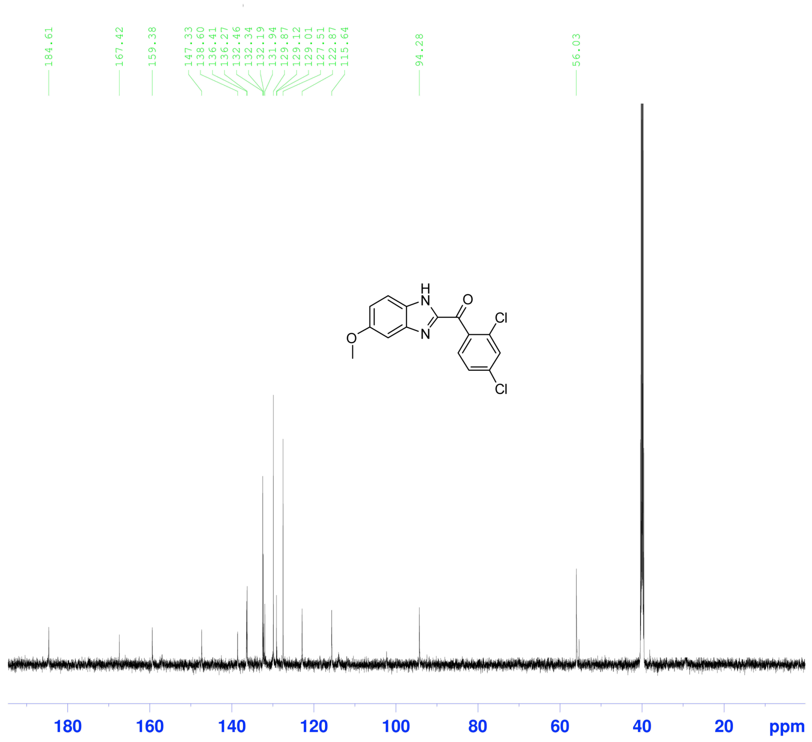

2-(4-Nitrol)benzoyl-5-methoxybenzimidazole

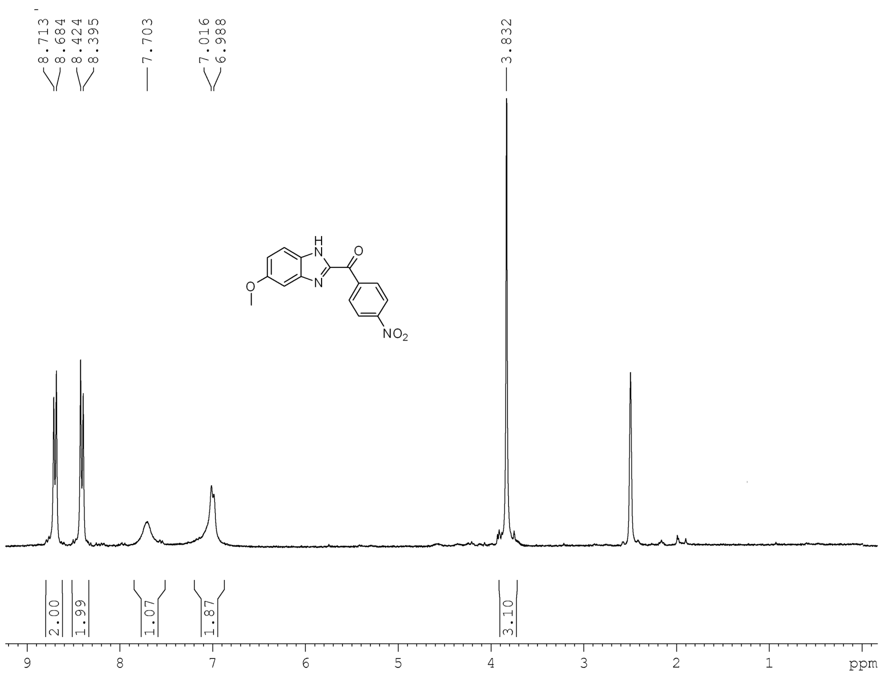

2-(4-Nitrol)benzoyl-5-methoxybenzimidazole

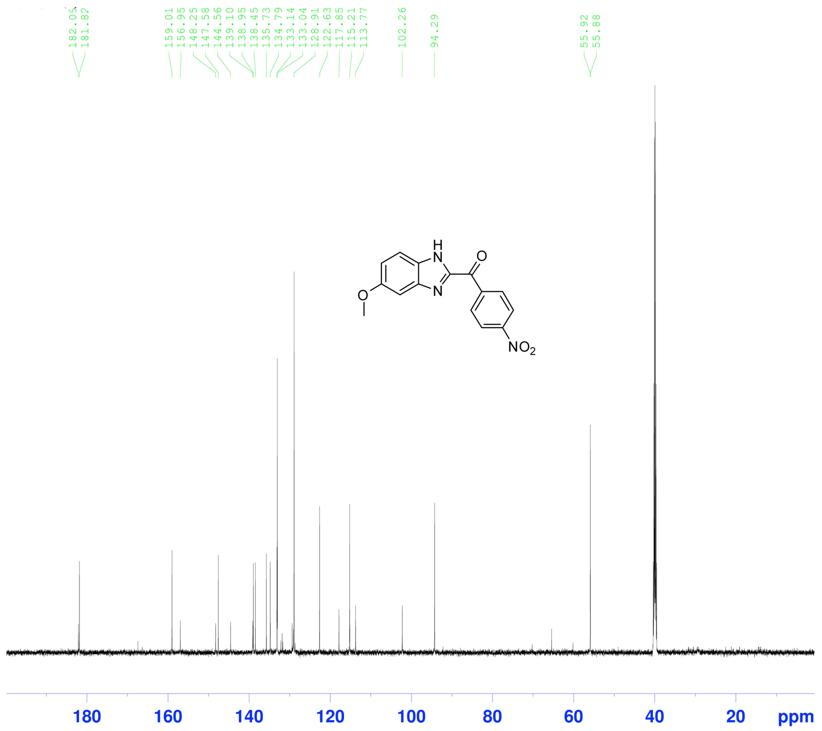

2-(3,4,5-Trimethoxy)benzoyl-5-methoxybenzimidazole

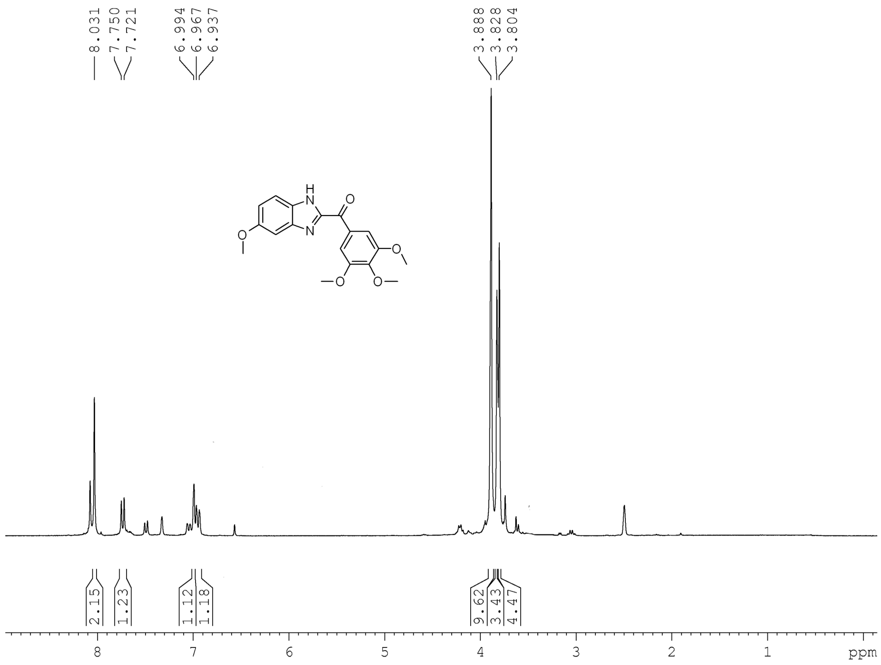

2-(3,4,5-Trimethoxy)benzoyl-5-methoxybenzimidazole

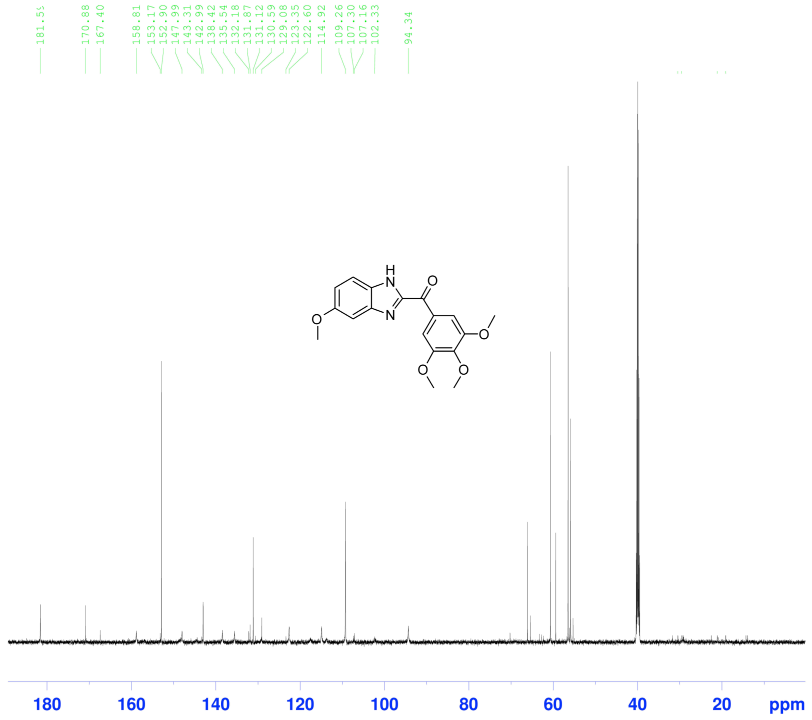

2-(4-Chloro)benzoyl-5-chlorobenzimidazole

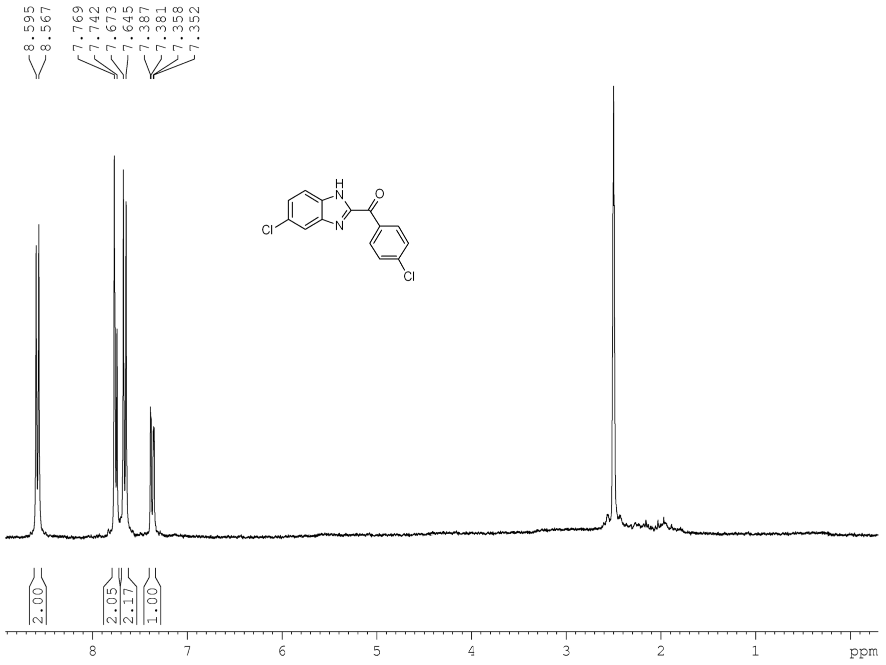

2-(4-Chloro)benzoyl-5-chlorobenzimidazole

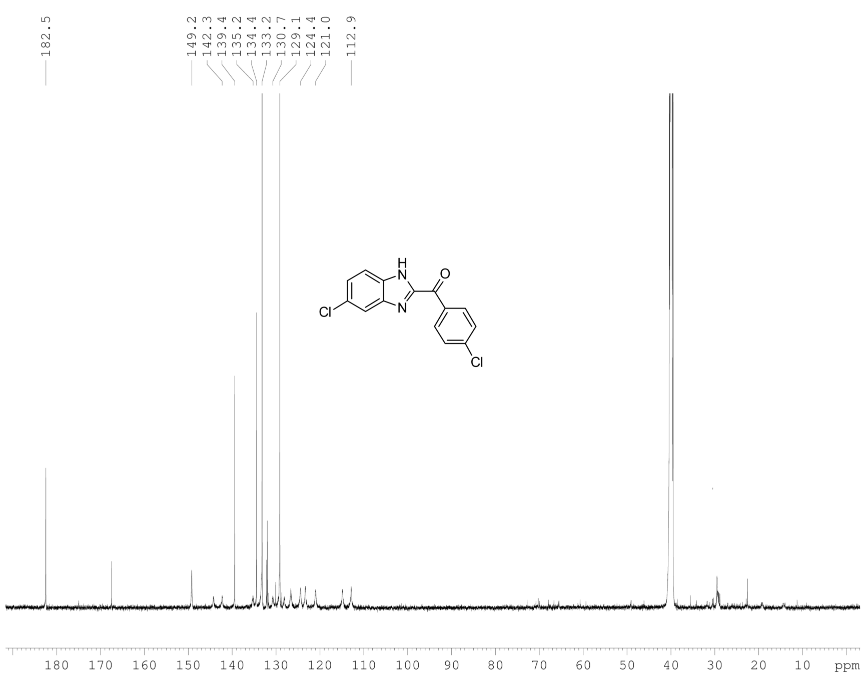

2-(2,4-Dichloro)benzoyl-5-chlorobenzimidazole

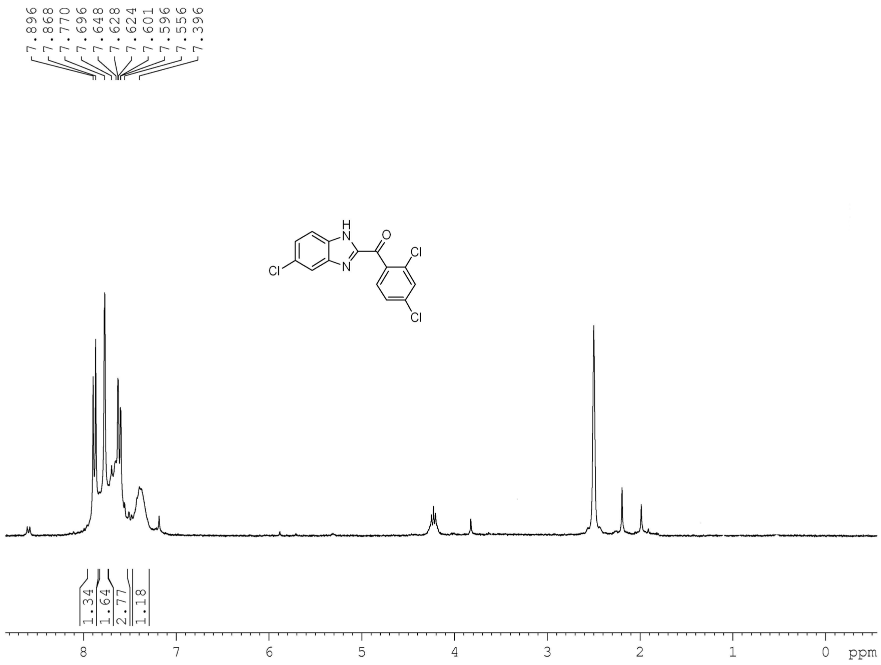

2-(2,4-Dichloro)benzoyl-5-chlorobenzimidazole

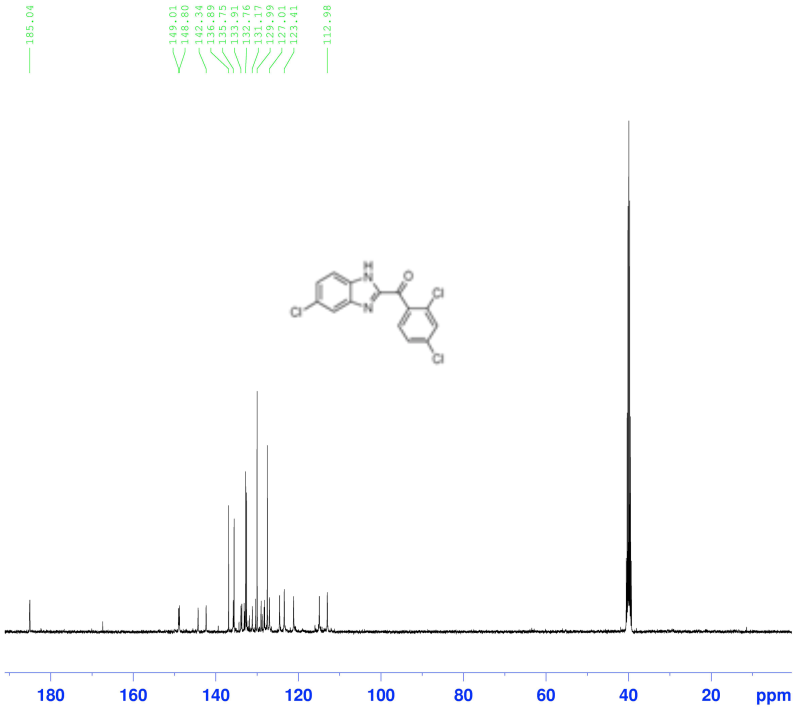

2-(4-Nitrol)benzoyl-5-chlorobenzimidazole

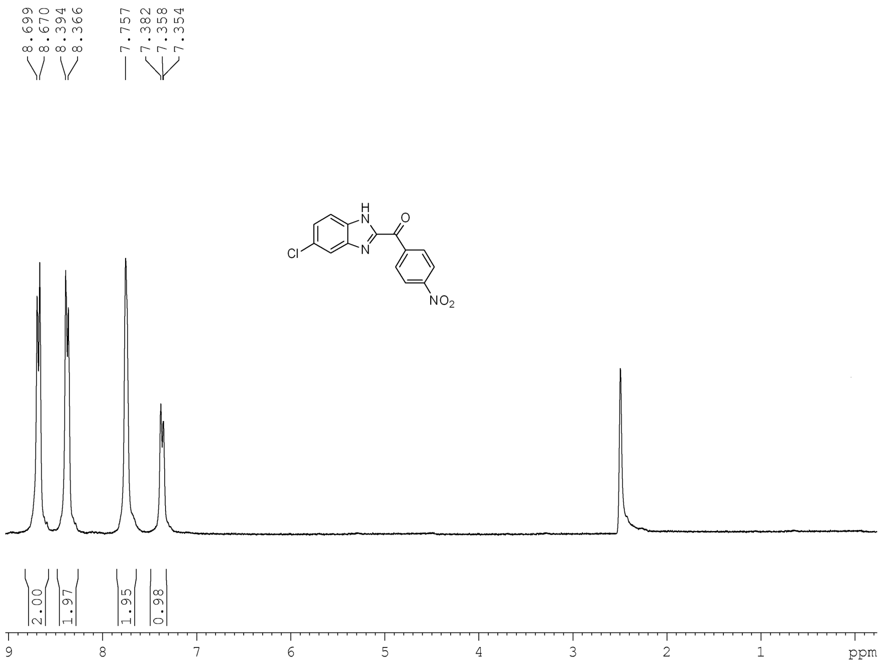

2-(4-Nitrol)benzoyl-5-chlorobenzimidazole

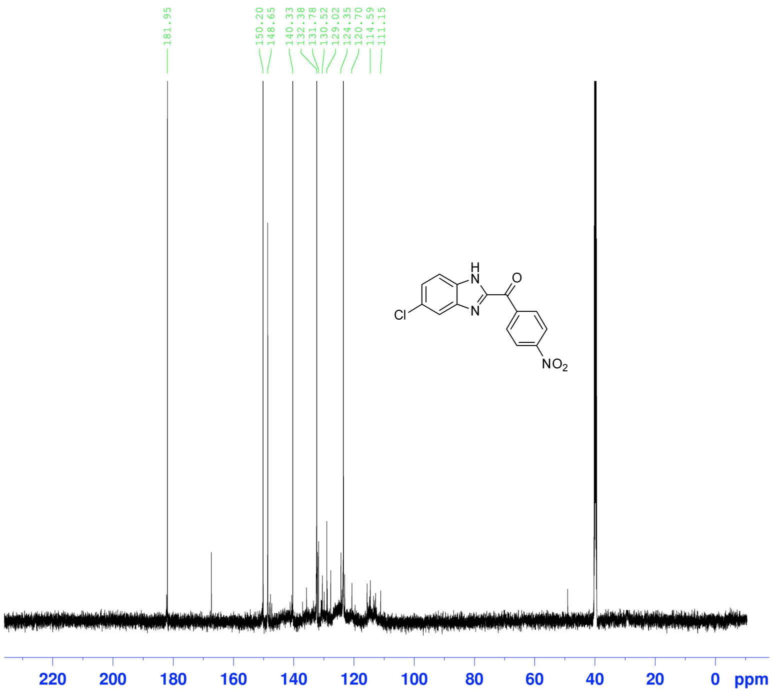

2-(3,4,5-Trimethoxy)benzoyl-5-chlorobenzimidazole

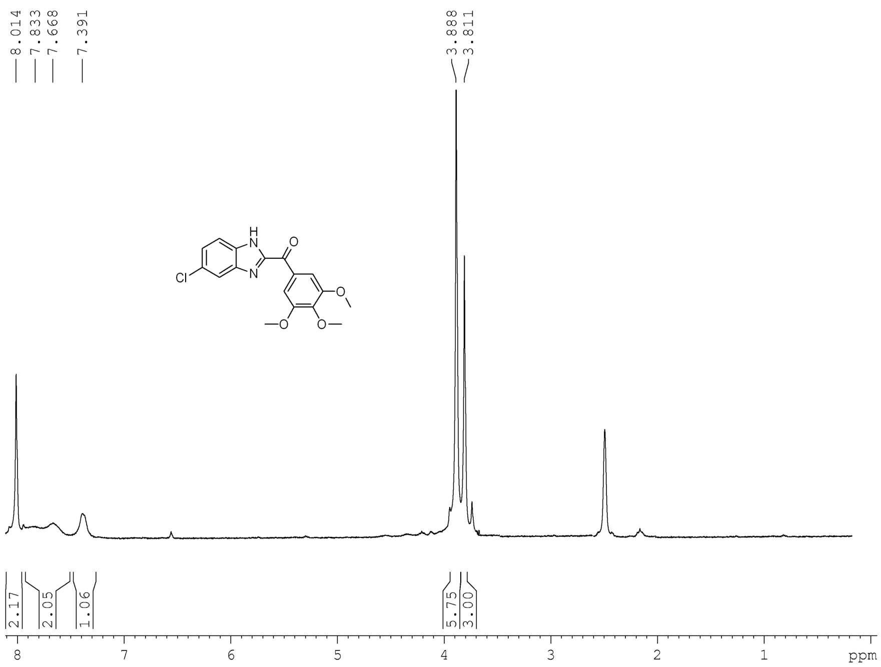

2-(3,4,5-Trimethoxy)benzoyl-5-chlorobenzimidazole

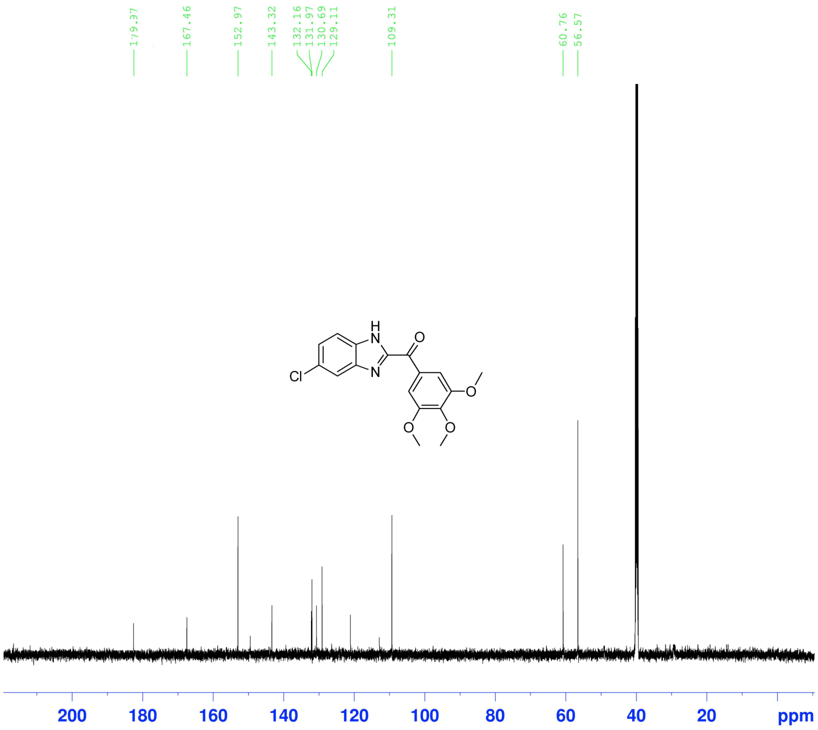

2-(4-Chloro)benzoylbenzoxazole

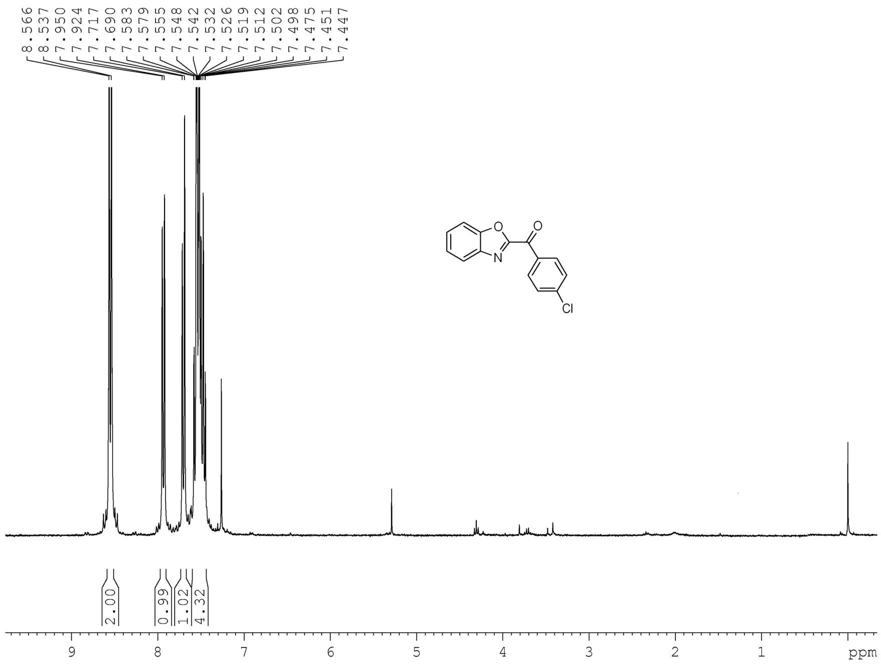

2-(4-Chloro)benzoylbenzoxazole

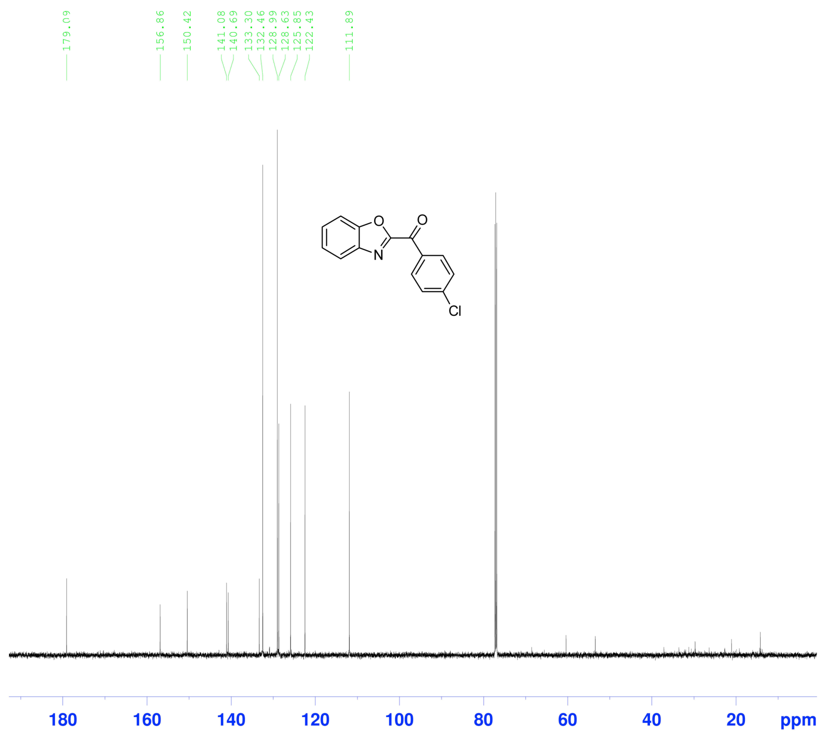

2-(2,4-Dichloro)benzoylbenzoxazole

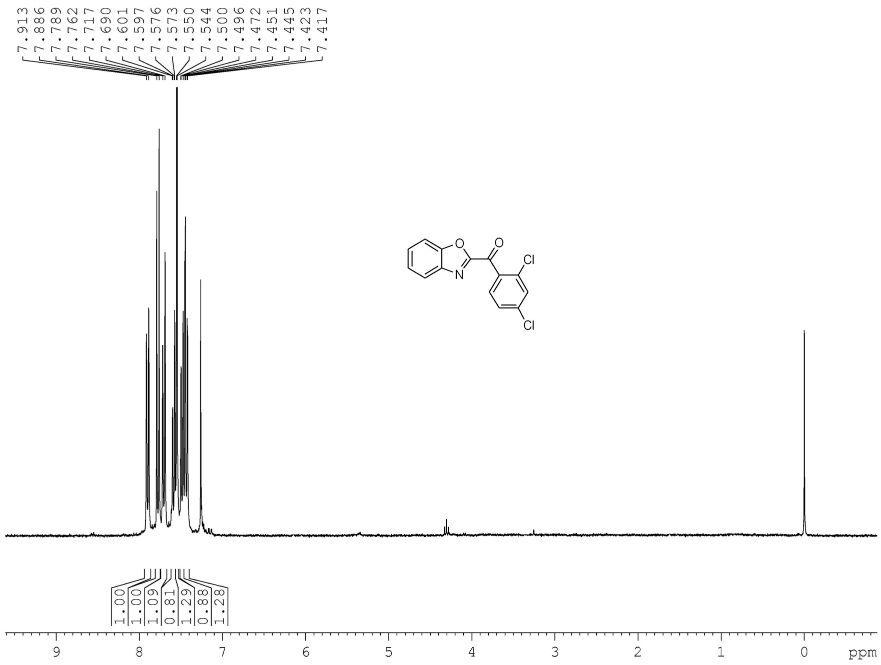

2-(2,4-Dichloro)benzoylbenzoxazole

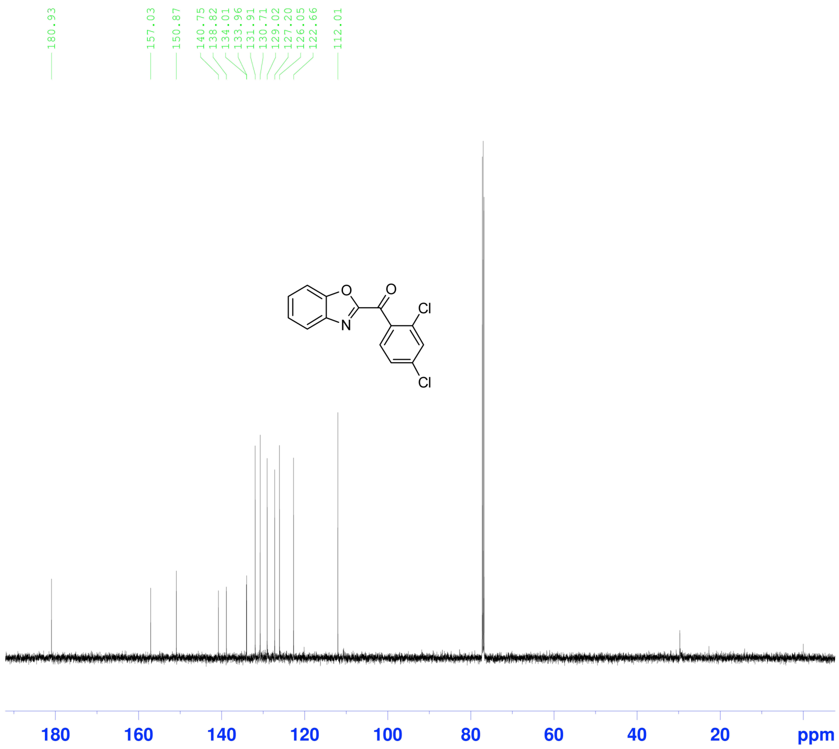

2-(4-Nitro)benzoylbenzoxazole

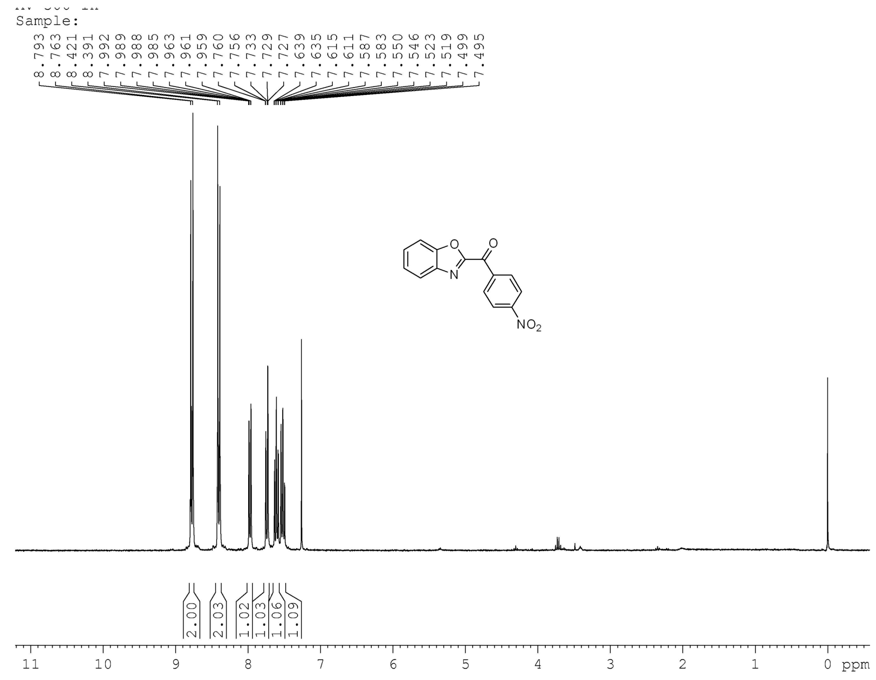

2-(4-Nitro)benzoylbenzoxazole

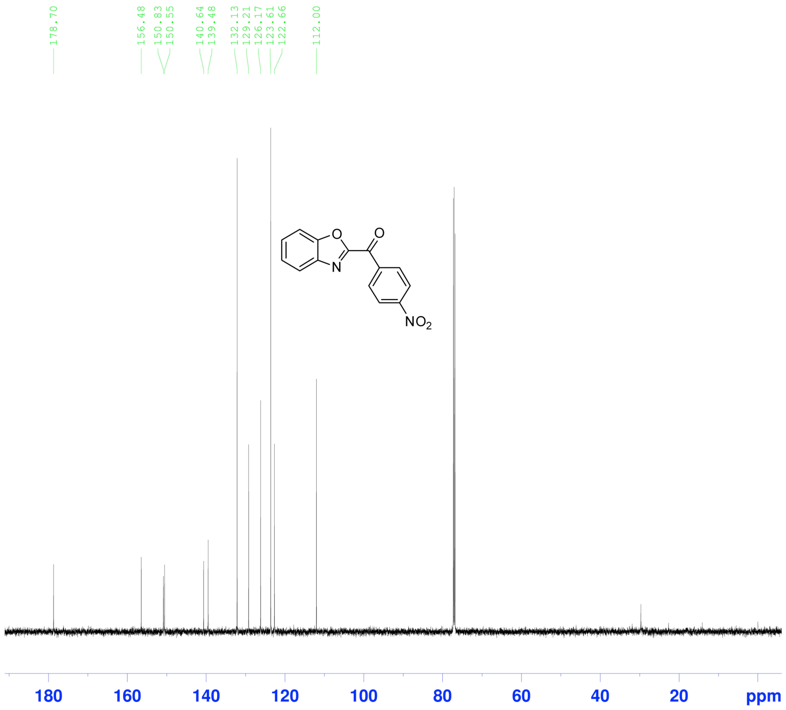

2-(3,4,5-Trimethoxy)benzoylbenzoxazole

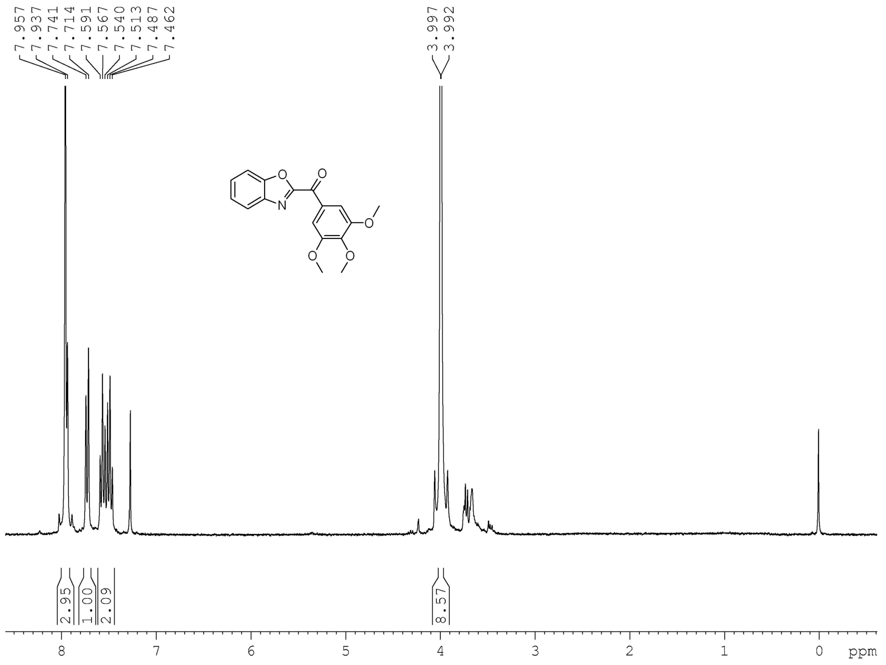

2-(3,4,5-Trimethoxy)benzoylbenzoxazole

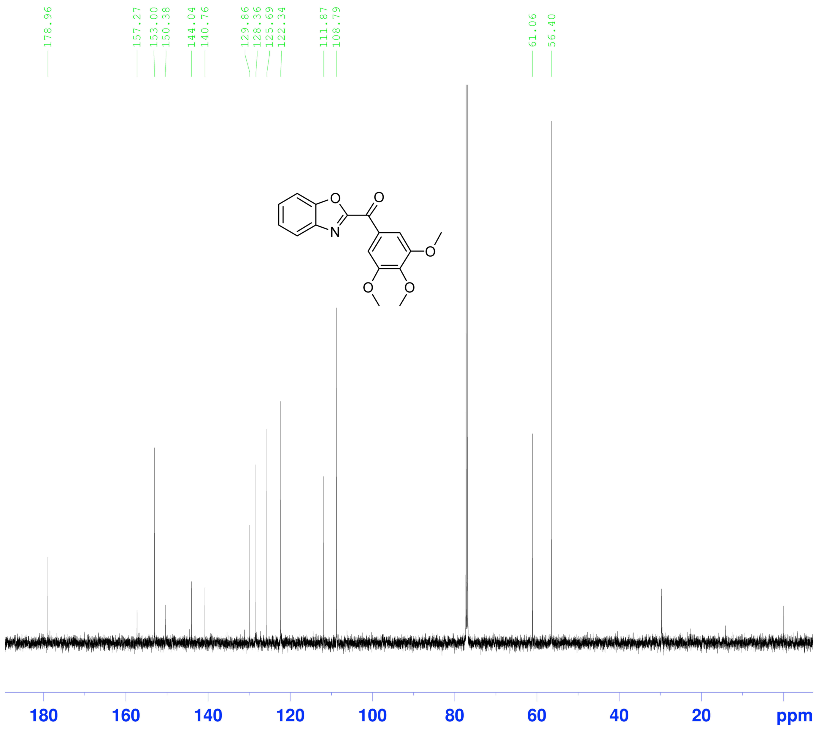

2-(4-Chloro)benzoyl-5-methylbenzoxazole

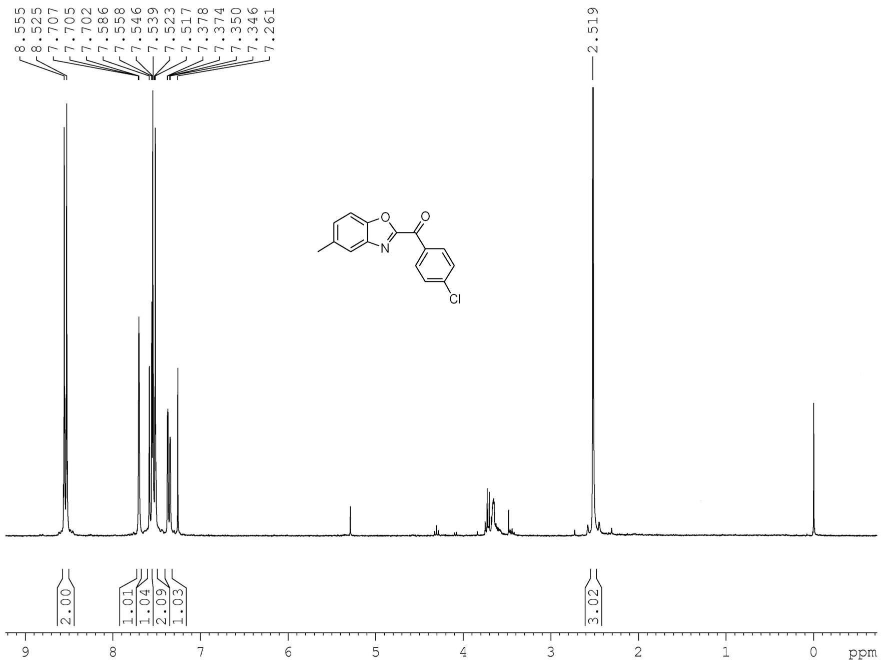

2-(4-Chloro)benzoyl-5-methylbenzoxazole

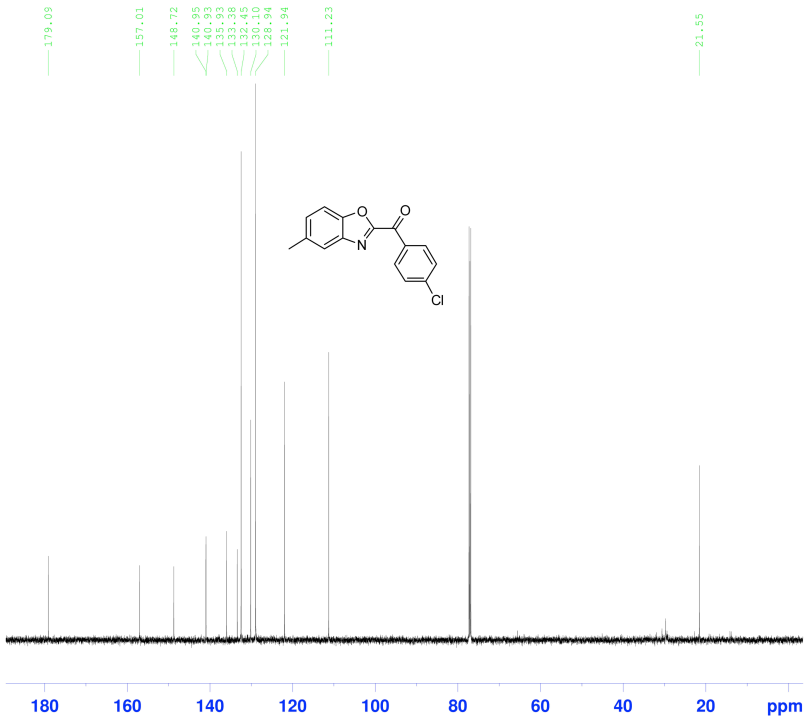

2-(2,4-Dichloro)benzoyl-5-methylbenzoxazole

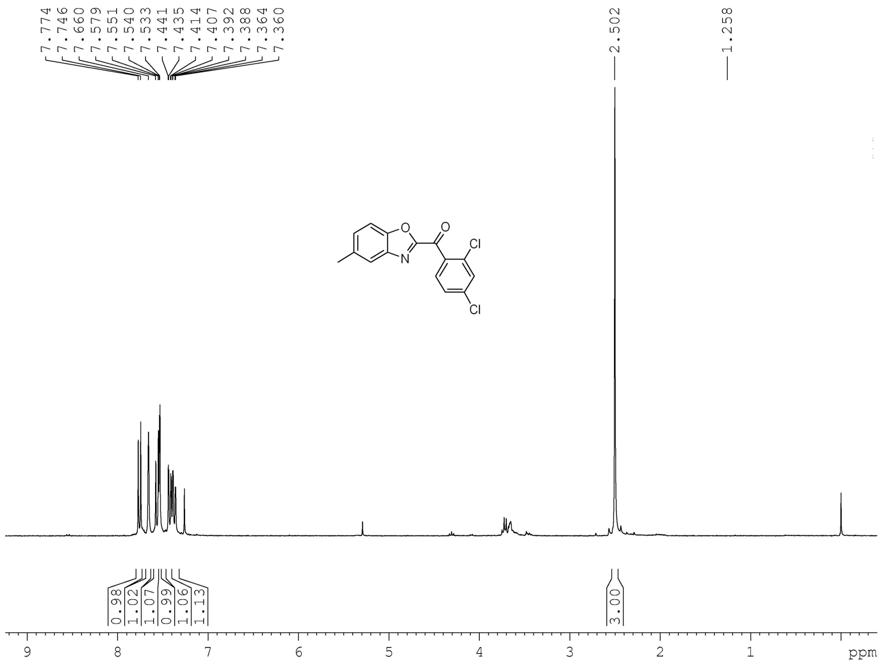

2-(2,4-Dichloro)benzoyl-5-methylbenzoxazole

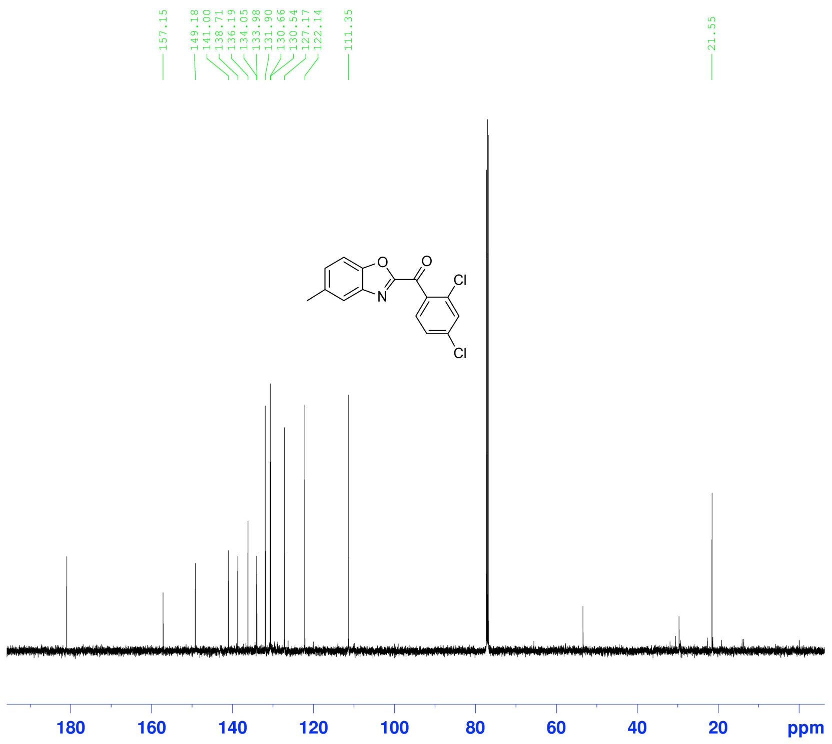

2-(3,4,5-Trimethoxy)benzoyl-5-methylbenzoxazole

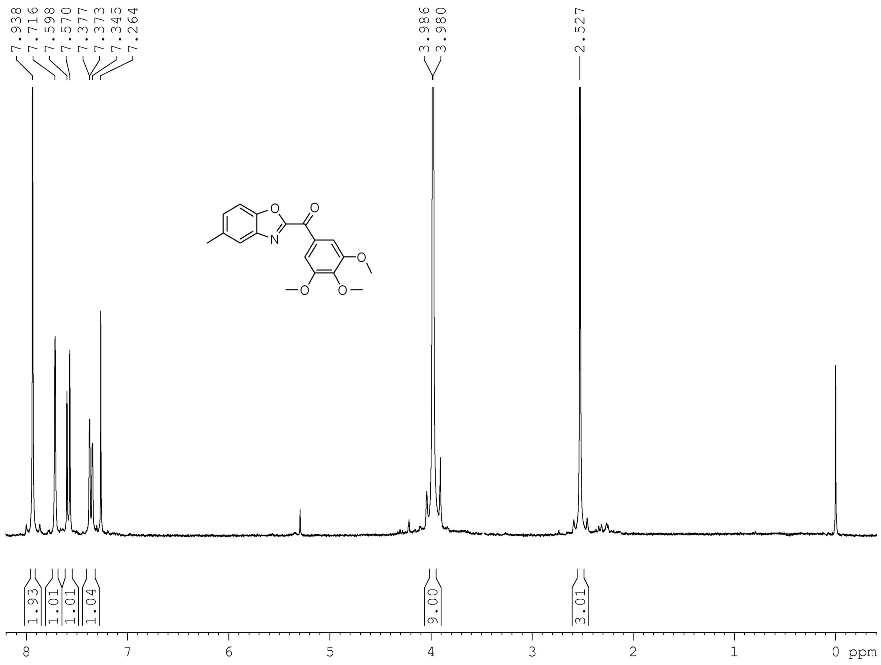

2-(3,4,5-Trimethoxy)benzoyl-5-methylbenzoxazole

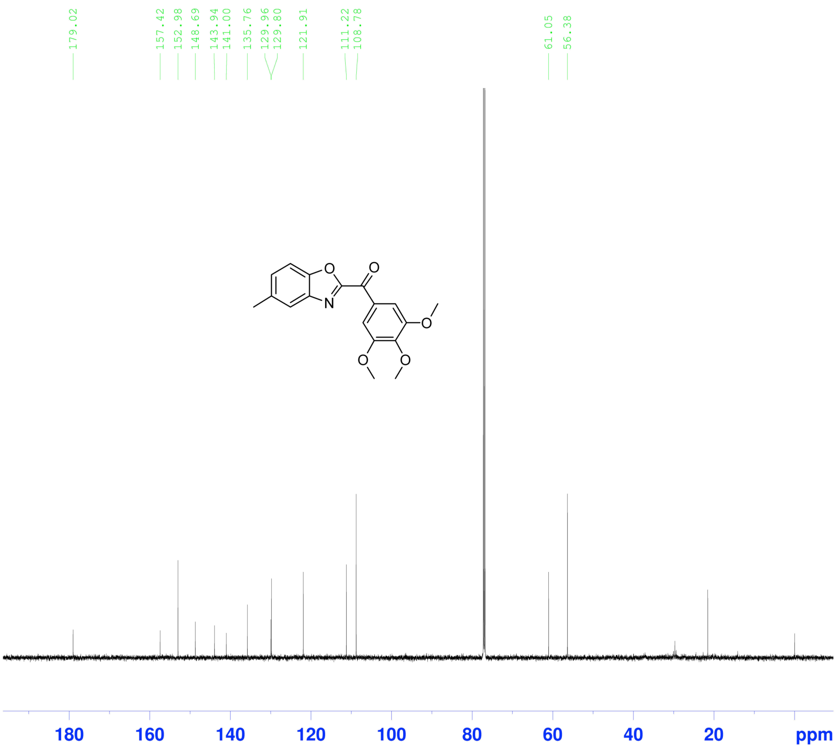

2-(4-Chloro)benzoyl-5-chlorobenzoxazole

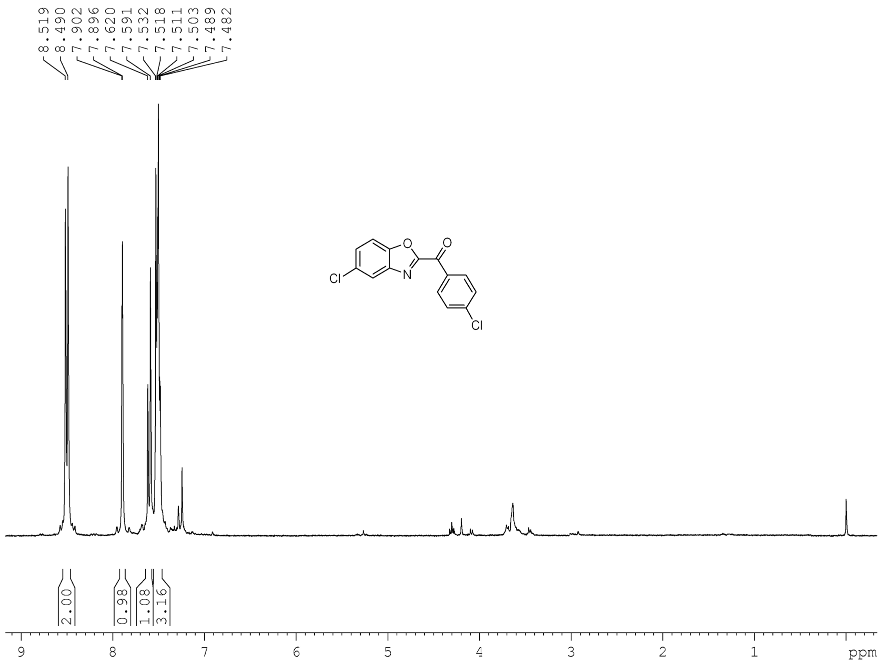

2-(4-Chloro)benzoyl-5-chlorobenzoxazole

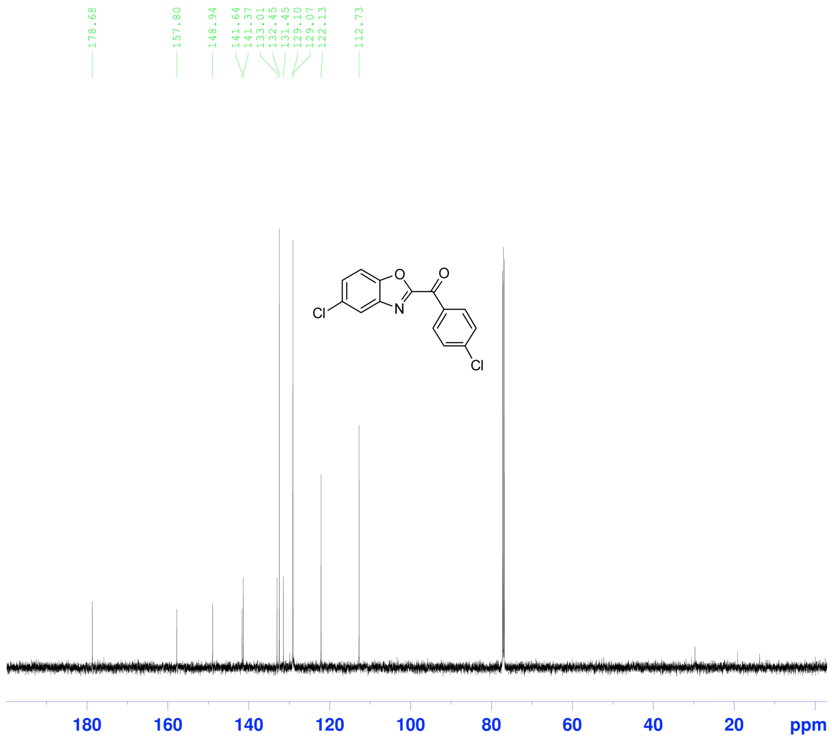

2-(3,4,5-Trimethoxy)benzoyl-5-chlorobenzoxazole

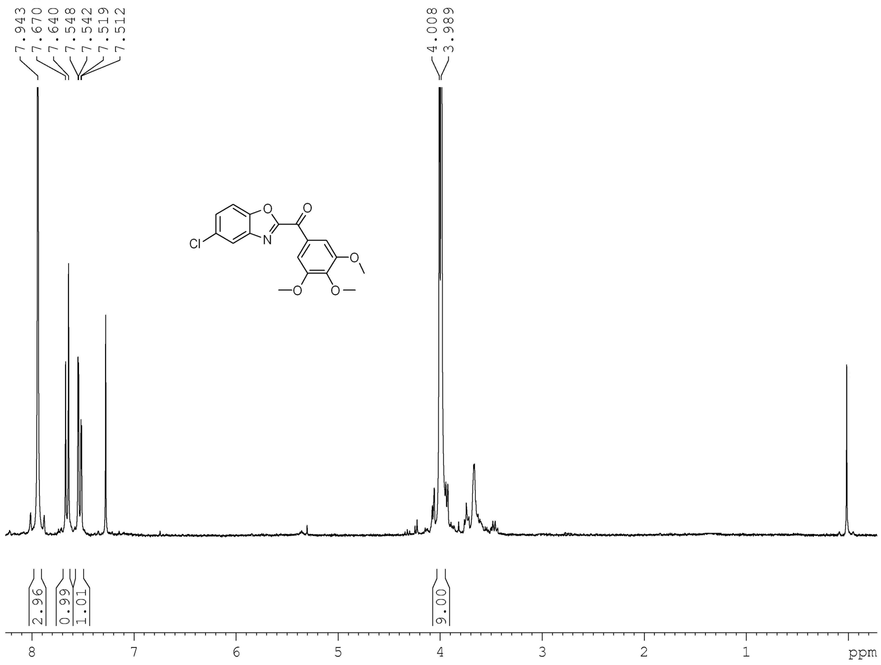

2-(3,4,5-Trimethoxy)benzoyl-5-chlorobenzoxazole

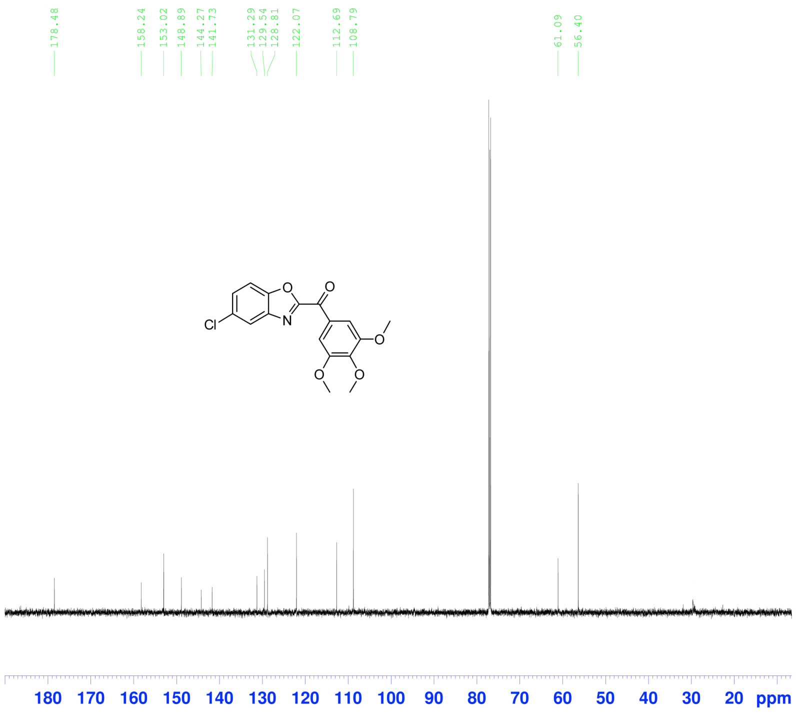

Supplement: Supplementary Information [file srep10360-s1.pdf]
